# Coupled geomorphic and climate-driven biogeochemical processes regulate soil organic carbon stocks in agricultural terraces

**DOI:** 10.1126/sciadv.aea8560

**Published:** 2026-02-25

**Authors:** Pengzhi Zhao, Daniel J. Fallu, Sebastian Doetterl, Sara Cucchiaro, Paolo Tarolli, Ben R. Pears, Andreas Lang, Moritz F. Mainka, Xiaojing Ou, Jeanette Whitaker, Zhengang Wang, Antony G. Brown, Johan Six, Kristof Van Oost

**Affiliations:** ^1^ Earth & Life Institute, Université catholique de Louvain, 1348 Louvain-la-Neuve, Belgium.; ^2^ UK Centre for Ecology Hydrology, LA1 4AP Lancaster, UK.; ^3^ UiT The Arctic University of Norway, 30, 9013 Tromsø, Norway.; ^4^ Department of Environmental Systems Science, ETH Zurich, 8092 Zürich, Switzerland.; ^5^ Department of Agricultural, Food, Environmental and Animal Sciences, University of Udine, 33100 Udine, Italy.; ^6^ Department of Land, Environment, Agriculure and Forestry, University of Padova, 35020 Legnaro, Italy.; ^7^ Geography and Environmental Science, University of Southampton, SO17 1BJ Southampton, United Kingdom.; ^8^ Department of Environment and Biodiversity, Paris Lodron University of Salzburg, 5020 Salzburg, Austria.; ^9^ Institute of Environmental and Ecological Engineering, Guangdong University of Technology, 510006 Guangzhou, China.

## Abstract

Agricultural terraces are among the most widespread human-made landforms. They profoundly reshape soil landscapes and influence the carbon cycle, yet the extent and drivers of their impact remain highly uncertain. By integrating field observations from 14 well-drained terrace landforms across a climatic-geochemical gradient with a data synthesis, we show that changes in soil organic carbon (SOC) stocks after terracing are governed by two coupled C turnover-geomorphic processes: replacement of lost topsoil C at eroding positions and stabilization of buried SOC at depositional positions. Climate strongly modulates these processes by shaping soil geochemistry and plant productivity, which in turn control SOC replacement and stabilization within terraces. Thus, terracing effects on SOC stocks shift from consistently positive in humid regions to mixed (positive and negative) outcomes in dry regions. This study establishes a framework for elucidating SOC dynamics in well-drained terrace systems and provides a scientific basis for targeted management strategies to enhance C sequestration in agricultural terraces globally.

## Teaser

Climate-driven biogeochemical processes explain why terracing can either enhance or deplete soil organic carbon stocks.

## INTRODUCTION

Agricultural terrace systems—being the volumetrically largest, most common, and often oldest artificial landforms—have been implemented globally to support essential soil ecosystem services, including erosion control, soil nutrient and water retention, and carbon (C) sequestration (*1*, *2*). The earliest terraces appeared over 5000 years ago in regions where agricultural civilization first emerged. Since then, terraces have been intimately linked to agriculture, spanning ~5% of global cropland areas (*3*, *4*); terraces cover one-third of China’s cropland (*5*), and in hilly regions of Greece, terraced area can even reach up to 70% of cropland (*6*). Hence, agricultural terraces are among the most extensive anthropogenic landscapes, second only to urban areas (*2*, *3*). Terracing practices have introduced extensive disturbances to topography, soil redistribution, and land use, thereby affecting soil organic carbon (SOC) storage and C exchange with the atmosphere (*1*, *7*–*10*). However, the direction and magnitude of this impact remain highly uncertain (*2*, *11*), with reported effects ranging from positive (up to 169% increase in SOC) (*8*, *9*, *11*) to negative (up to 72% decrease in SOC) (*7*, *12*–*14*), and the underlying factors driving these diverging impacts are unknown. A comprehensive understanding of the net effect of terracing on SOC stock and the underlying processes is therefore urgently needed to assess the potential role of rapidly expanding terraced land in SOC sequestration and climate change mitigation.

Terraces are typically constructed by excavating soil from the upper slope (hereafter cut position; fig. S1) and redistributing them into the lower position of slope (hereafter fill position). Alternatively, terraces are formed through soil erosion, deposition, and cultivation and are commonly known as lynchets (*1*, *2*). Terrace construction establishes a geomorphic template via soil redistribution, modulating biogeochemical processes that drive SOC dynamics. Construction exposes subsurface soils in the cut/erosion positions of the terraces and buries original topsoil in the fill/deposition positions (fig. S1). The removal of C-rich topsoil in the cut/erosion positions depletes SOC during the early stages of terrace formation (*7*), followed by lost SOC replacement because of plant C inputs and soil’s capacity to stabilize fresh organic C. The magnitude of plant C input (i.e., roots, litter, and exudates), soil physicochemical properties, and terrace age primarily control the SOC replacement rate (*8*, *15*). In the fill/deposition position of terraces, the burial of original topsoil leads to SOC accumulation, with its stability dependent on environmental conditions (e.g., temperature and moisture) and mineralogical properties relevant to SOC persistence (*9*, *16*, *17*). Because climate controls not only net primary productivity (NPP) (*18*), hence plant C inputs to soil, but also mineral weathering and the abundance of reactive mineral surfaces, hence SOC stabilization (*19*–*21*), soil, plant, and climatic properties are tightly interconnected. At present, however, a comprehensive study that fully captures the climatic, pedogenic, and vegetative controls on SOC stock change in terrace systems is lacking (*8*, *9*, *22*), particularly when considering the growing awareness of geomorphic controls on SOC turnover (*23*).

To address these knowledge gaps, this study aims to (i) identify the primary controls underlying the substantial variability in SOC stock responses to terrace construction reported in previous studies (*7*–*9*, *11*–*14*) and (ii) elucidate the mechanisms by which these controls regulate SOC stock change due to terrace implementation. These efforts seek to provide a science-based assessment of the role that widely implemented agricultural terraces may play in SOC sequestration and climate change mitigation. To this end, we investigated 14 agricultural terrace landforms distributed along a climatic-geochemical gradient across Europe (table S1). The sampling sites span semi-arid, semi-humid, and humid climate zones, broadly representing the major climatic regions where terraces are globally distributed [see map from (*4*) and Materials and Methods]. First, we compared SOC stocks (down to bedrock) between terraced and nonterraced sloping landforms across 14 study sites, revealing climate as the primary driver of the pronounced variability in SOC stock changes due to terracing across regions. We then validated the generality of this finding through a synthesis of 99 paired datasets on SOC changes due to terracing from 58 publications, which confirmed the dominant role of climates. Last, using 485 depth-explicit soil samples collected from 14 terrace sites along the climatic-geochemical gradient, we analyzed 26 soil physical and geochemical properties—together with geomorphic features, plant productivity, terrace age, and climatic variables—to unravel how climatic-geomorphic factors govern the magnitude and direction of SOC stock change following terracing (see Materials and Methods).

## RESULTS AND DISCUSSION

### Key drivers shaping the magnitude and direction of terracing effects on SOC stocks

**Fig. 1.**
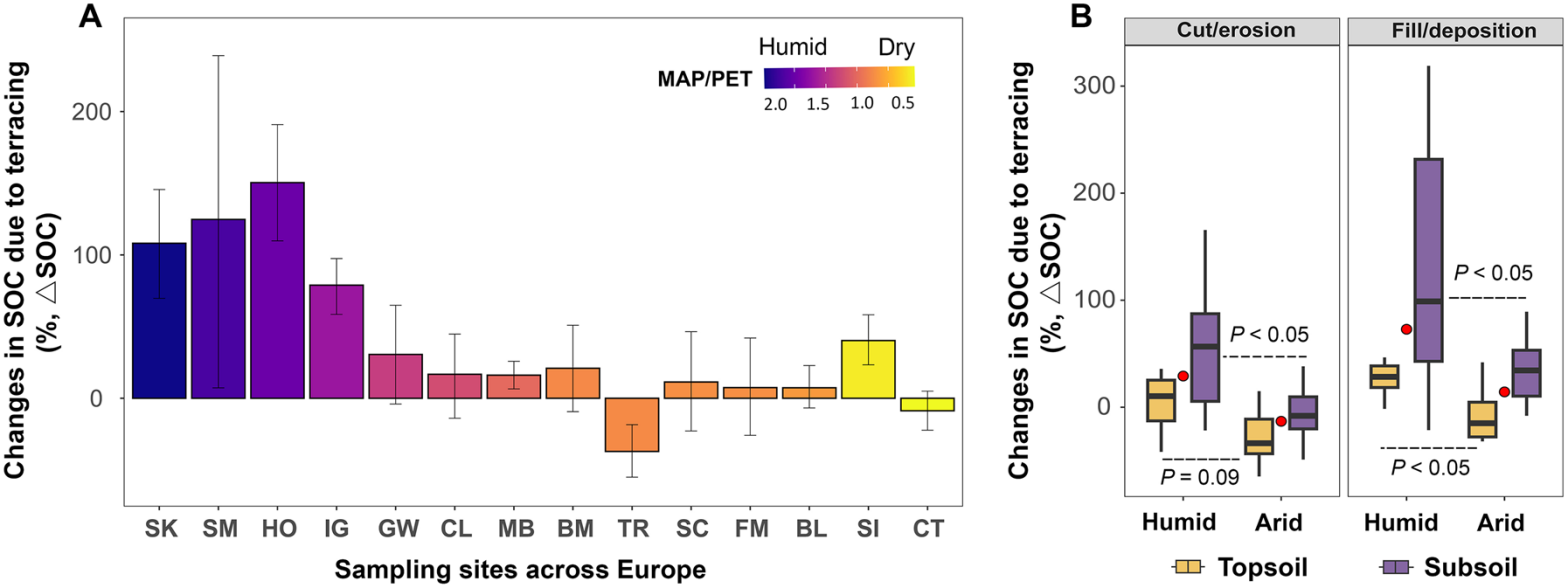
Terrace-induced changes in SOC stock (∆SOC) and its relationships with climates and geomorphic positions. (**A**) ∆SOC (means ± 95% confidence intervals) estimated from 14 study sites across Europe down to the weathered rock surfaces. Sample site initials resolved in table S1. $\text{∆SOC}=100\times \left[\left(\text{SOC}\;{\text{stock}}_{\text{terraced}}-\text{SOC}\;{\text{stock}}_{\text{nonterraced}}\right)/\text{SOC}\;{\text{stock}}_{\text{nonterraced}}\right]$. Colors represent the aridity index (AI) (MAP/PET), where the dashed line indicates humid (MAP/PET > 1) versus arid (MAP/PET < 1) regions. (**B**) Comparison of ∆SOC between slope positions and soil depth layers (topsoil, <15 cm; subsoil, >15 cm) of terrace sequences. The red points are the average of ∆SOC for all soil layers. Box plots represent first and third quartiles (box), medians (central horizontal line), upper whisker (upper vertical line), and lower whisker (lower vertical line). *P* < 0.05 indicates a significant difference in ΔSOC at a given slope position between humid and arid regions.

The results revealed considerable variability in both the direction and magnitude of terracing effects on SOC stock. Compared to nonterraced profiles (hereafter control), terracing led to an average increase of 41 ± 15% [hereafter mean ± standard error (SE) unless otherwise stated] in SOC stock (down to bedrock, mean soil depth = 94 cm) across 14 study sites (Fig. 1A). This value is comparable to that reported at the national scale (e.g., 32%) (*11*). However, responses were not uniform: Terracing significantly enhanced SOC stocks at most sites (*N* = 12), whereas a few sites (*N* = 2) showed minimal change or even SOC depletion, consistent with previous studies reporting both positive and negative SOC stock responses to terracing across regions (*7*–*9*, *11*–*14*).

**Fig. 2.**
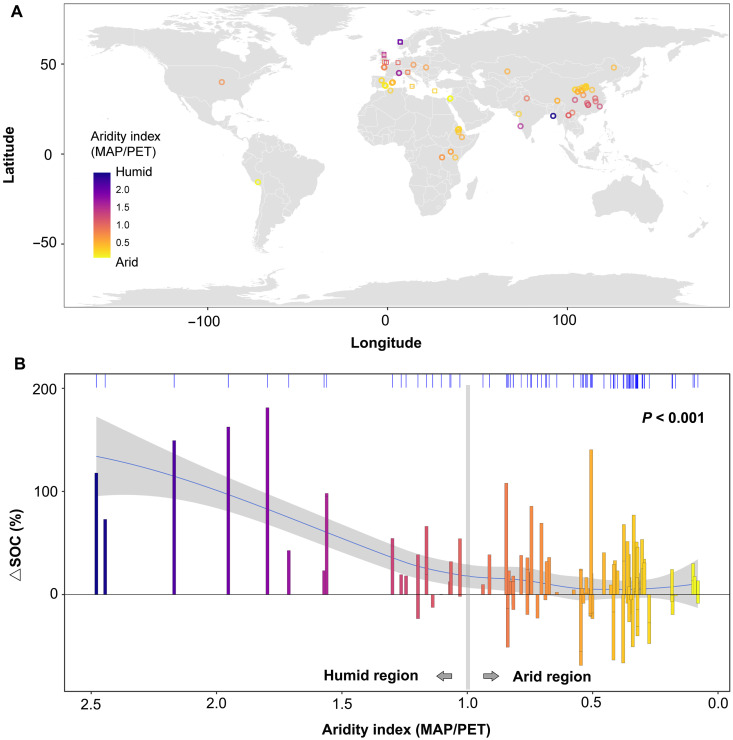
Large-scale validation of the pronounced role of climate in explaining divergent SOC responses to terracing. (**A**) Distribution of datapoints (*N* = 144) across globe: Circles represent the dataset extracted from 58 independent published studies, while squares represent paired data from the European survey. (**B**) Changes in SOC due to terracing (∆SOC, %) and its relationships with climate conditions at the global scale. Colors represent the AI (MAP/PET). The loess/spline smooth line (blue line) indicates the overall trend (*P* < 0.001). The gray region around the smooth line indicates the confidence interval at 95%. The marginal upper axis tick (*x* axis) marks illustrate the distribution of datapoints along AI.

Our data showed that this variability in SOC responses to terracing was closely associated with climate conditions. In relatively humid regions [mean annual precipitation (MAP)/potential evapotranspiration (PET) > 1, hereafter humid], SOC stock change after terracing relative to nonterraced controls (hereafter ∆SOC) were strongly positive (75 ± 21%). In contrast, in relatively dry regions (MAP/PET < 1, hereafter arid), ∆SOC was smaller (6 ± 9%) and ranged from positive (40%) to negative (−37%) (Fig. 1A), indicating the inconsistent effect of terracing on SOC stock under arid conditions. This is further supported by a linear regression showing that climates (MAP/PET) was a primary control on ∆SOC [coefficient of determination (*R*^2^) = 0.61, *P* < 0.001; fig. S2]. The importance of climate was further validated by our large-scale SOC data synthesis (Fig. 2 and Materials and Methods; average soil depth = 54 cm), which revealed a clear shift from consistently positive to mixed (both positive and negative) SOC stock responses to terracing as climatic conditions transitioned from wet to dry (MAP/PET = 1). Thus, both field observations and additional SOC data synthesis conclusively demonstrate that the apparent paradox of terracing-induced SOC stock changes—whether agricultural terrace implementation enhances or depletes SOC stocks (*7*–*9*, *11*–*14*)—can be largely resolved by the pivotal influence of climate conditions (Fig. 2).

### Processes mediating climate-driven divergent terracing-induced SOC changes

To provide a mechanistic explanation for the observed climate-driven divergence in terracing-induced SOC stock changes (i.e., Figs. 1 and 2), we investigated the geomorphic process, plant productivity, and soil geochemical factors underlying these differences, drawing on soil samples collected from 14 terrace sites along a climatic-geochemical gradient across Europe (see Materials and Methods). These factors were selected because they were strongly influenced by climate and play critical roles in regulating SOC dynamics, as documented in previous studies (*19*, *20*, *23*, *24*).

Terrace construction redistributes soil through erosion and deposition, creating a distinct geomorphic template that governs SOC dynamics (fig. S1) (*8*, *25*). Here, we compared ∆SOC between terraces’ geomorphic positions under different climate conditions to assess how terracing-induced soil redistribution interacts with climate to drive divergent SOC changes within the terrace system. In the fill/deposition positions where topsoil burial and accumulation occur during terrace establishment (fig. S1), the average ∆SOC in humid regions was significantly higher than in arid regions (Fig. 1B). This suggests that the SOC accumulation due to topsoil burial during terrace establishment is significantly higher in humid than arid regions. In the cut/erosion positions with notable topsoil loss during the early stage of terrace establishment (fig. S1), terraces in humid regions showed an increase in SOC stock relative to the control (∆SOC = 38 ± 13%; Fig. 1B), whereas those in arid regions showed a decline in SOC stock (∆SOC = −12 ± 8%). These results indicate that the lost topsoil C at the cut/erosion positions due to terrace excavation has been completely replaced in humid regions, but not in arid regions. Furthermore, the subsoil layers (i.e., below 15 cm) showed significantly higher ∆SOC than the topsoil layers (<15 cm) at both slope positions of terraces (*P* < 0.05), indicating that the ∆SOC is mainly driven by geomorphic processes occurring in the subsoil layers (Fig. 1B). These results suggest that regional climates modules the coupled SOC turnover-geomorphic processes occurring at cut/erosion and fill/deposition position of terraces, leading to significantly greater ∆SOC in humid regions than in arid regions.

**Fig. 3.**
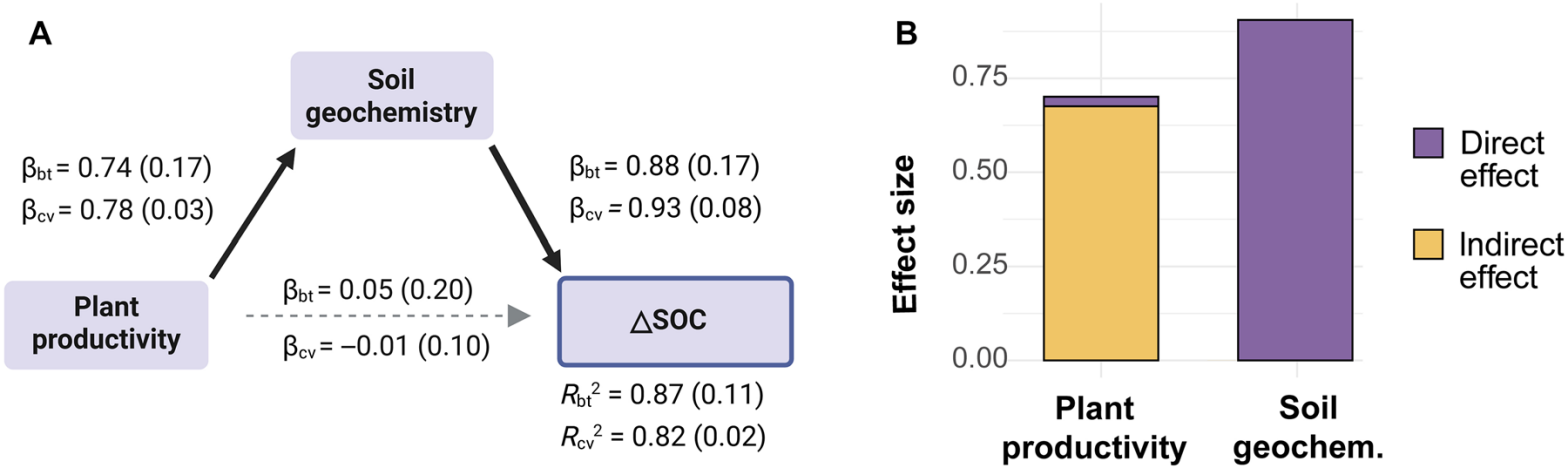
Effect of TPP and soil geochemistry on terracing-induced changes in SOC stock (∆SOC) estimated by PLS-SEM analysis. The numbers between the latent variables in (**A**) represent the standardized path coefficients based on bootstrapping (β_bt_) and 10-fold cross-validation (β_cv_), with SDs shown in parentheses. Solid and dashed arrows indicate significant (*P* < 0.05) and nonsignificant (*P* ≥ 0.05) coefficients, respectively*. R*_bt_ and *R*_cv_ denote the coefficients of determination (*R*^2^) derived from bootstrapping and 10-fold cross-validation, respectively. (**B**) Total effect (including direct and indirect effects) of plant productivity and soil geochemistry on ∆SOC.

To assess the effect of climate-dependent soil development and geochemistry on terracing SOC stock, a wide range of soil physicochemical properties (26 variables) were measured on soil samples collected from 14 terrace sites (see Materials and Methods). To assess the potential impact of terracing-induced changes in land management and soil conditions on SOC stocks, we used an integrative proxy—the difference in current total plant productivity (TPP) between terraced and nonterraced control sites (hereafter ∆TPP) (see Materials and Methods). We assume that the observed current ∆TPP reflects the cumulative long-term ecological effects of terracing—such as changes in land management, erosion control, nutrient retention, and soil fertility—which ultimately lead to changes in plant productivity and, consequently, C inputs to the soil (*1*, *2*). Based on the partial least squares structural equation modeling (PLS-SEM; see Materials and Methods), we found that soil geochemistry and ∆TPP jointly explained 83 to 87% of the variance in ∆SOC (Fig. 3A). The total effects of soil geochemistry (effect size = 0.9) and ∆TPP (effect size = 0.7) on ∆SOC were broadly comparable. Soil geochemistry had a strong direct impact on ∆SOC (β = 0.91 to 0.92), while the effect of ∆TPP was predominantly indirect, mediated through soil geochemistry (β = 0.68 to 0.71) (Fig. 3). This finding was further supported by partial correlation analysis, which revealed that controlling for soil geochemistry significantly changed the zero-order correlation between plant productivity and ∆SOC (reduced from 0.70 to −0.02; fig. S3). These results indicate that SOC stock changes during and after terrace construction are associated with the interaction between soil geochemistry and plant productivity, both widely recognized as being strongly influenced by climatic conditions.

Collectively, our results outline a mechanistic framework, showing that the magnitude and direction of terracing effects on SOC stocks are governed by two coupled C turnover-geomorphic processes: (i) replacement of lost topsoil C at cut/erosion positions and (ii) stabilization of buried SOC at fill/deposition positions. These processes are strongly modulated by climate, which controls how plant productivity and soil geochemical properties interact to regulate SOC replacement and stabilization within terrace systems. Building on this framework, we then used 485 depth-explicit terraced soil samples from 14 terrace sites along a climatic-geochemical gradient to elucidate how climate conditions, in conjunction with geomorphic processes, modulate the role of soil geochemical properties and plant productivity in regulating SOC accumulation and loss across terrace systems, with detailed analyses of these factors in the following subsections.

### Effect of soil geochemistry

**Fig. 4.**
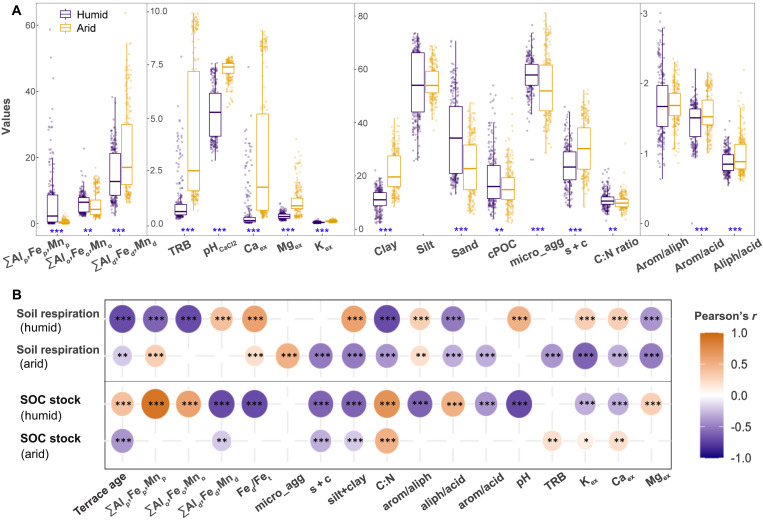
Terraced soils from 14 study sites along a climate gradient (*n* = 485) differ in their physiochemical properties relevant to SOC stabilization. (**A**) Comparison of soil physiochemical properties in terraces between humid and arid climate categories. Boxplots represent first and third quartiles (box), medians (central horizontal line), upper whisker (upper vertical line), and lower whisker (lower vertical line). (**B**) Correlations between SPR (μgC h^−1^ gSOC^−1^), SOC stock (kg C m^−2^ cm^−1^_depth_), and soil physiochemical properties. Color indicates the strength and sign of the correlation. Significant differences are indicated as follows: *P < 0.05, **P < 0.01, and ***P < 0.001. Blanks indicate insignificant relationships (*P* > 0.05). ΣAl_p_, Fe_p_, Mn_p_ = sum of organically complexed Al, Fe, and Mn oxides (g/kg); ΣAl_o_, Fe_o_, Mn_o_ = sum of poorly crystalline Al, Fe, and Mn oxides (g/kg); ΣAl_d_, Fe_d_, Mn_d_ = sum of highly crystalline Al, Fe, and Mn oxides (g/kg); Fe_d_/Fe_t_ = ratio of crystalline Fe oxide to total Fe. micro_agg = microaggregated OC (from 53 to 250 μm, % of total C); s + c = free silt and clay associated OC (<53 μm, % of total C). clay, silt, and sand contents are in %; arom/aliph = ratio of aromatic to aliphatic compound; aliph/acid = ratio of aliphatic to protonated COOH compounds, arom/acid = ratio of aromatic to protonated COOH compounds. TRB = total reserve in bases (cmol/kg). K_ex_, Ca_ex_, Mg_ex_ = exchangeable cations of K^+^, Ca^2+^, and Mg^2+^ (cmol/kg).

**Fig. 5.**
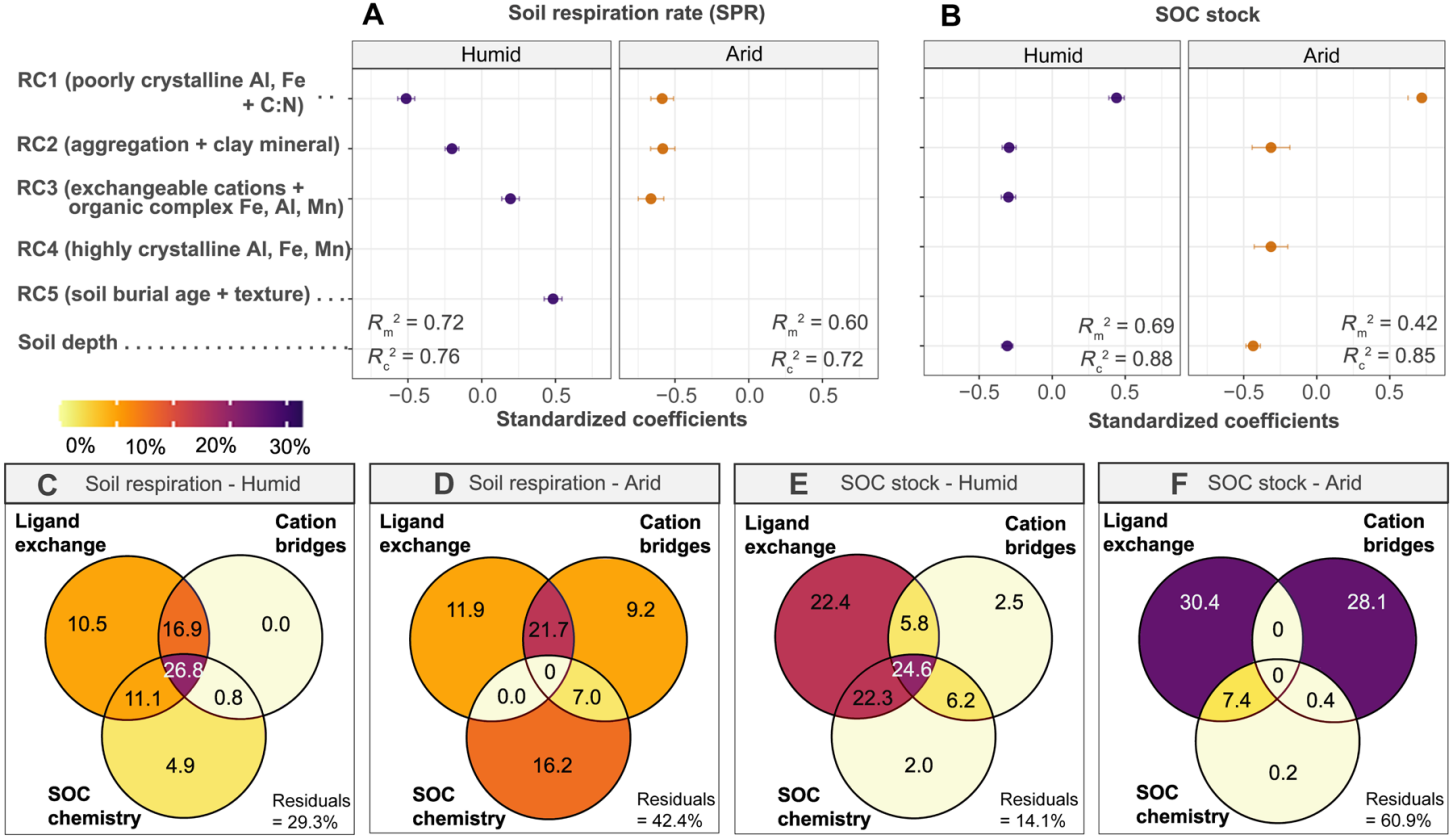
The dominant SOC stabilization mechanism in terraced soils differed between humid versus arid regions. (**A** and **B**) Results of linear mixed-effects models and relative importance analysis (standardized coefficients). Soil potential respiration rate and SOC stock were explained by soil depth and five retained components (RC1 to RC5) after rotated principal components analysis (rPCA) analysis. Results of rPCA are given in table S5. Fixed effects are indicated by marginal *R*^2^ (*R*^2^_m_), while conditional *R*^2^ (*R*^2^_c_) accounts for both fixed and random effects. (**C** to **F**) Variation partitioning analysis for SPR and SOC stock. The total variation is portioned into three primarily SOC protection mechanism: Ligand exchange, cation bridging, and SOC chemistry (see details in Materials and Methods). Numbers in overlapping area of two circles are the shared effects of two factors and, in each circle, are the unique effects of the corresponding factor.

In humid climates, higher moisture availability supports a higher degree of mineral weathering and depth distribution of secondary weathering products (*19*, *24*), resulting in acidic soils abundant in reactive metal oxides, i.e., organically complexed (ΣAl_p_, Fe_p_, Mn_p_) and poorly crystallized (ΣAl_o_, Fe_o_, Mn_o_) oxides (i.e., Fig. 4). These reactive metal oxides offer dense hydroxyl sites that form covalent metal–O–C bonds via rapid ligand-exchange reactions with the carboxyl or hydroxamate groups present in organic matter (*26*, *27*)*.* Organic matter stabilized via this pathway can persist over the long term and has been proposed as one of the energetically strongest SOC protection pathways (*28*). As a result, we observed significant correlations between both SOC stocks and soil respiration of terraced soils with reactive metal oxides in humid climates (Fig. 4), establishing a strong SOC protection pathway (e.g., ligand exchange in humid terraces). This pathway was revealed by the linear mixed-effect (*R^2^* = 0.42–0.72; Fig. 5, A and B) and nonlinear models (*R*^2^ = 0.70 to 0.93; fig. S4; see Materials and Methods). Linear mixed-effect models revealed that, for terraces in humid regions, the most powerful predictor for soil potential respiration (SPR) was a component (RC1) representing poorly crystalline Al, Fe oxides, and SOC C:N ratio (Fig. 5, A and B). The nonlinear models confirmed that for terraces in humid regions, reactive Fe oxides ranked as the top variables controlling SPR (Fe_o_), and SOC stock (Fe_p_; fig. S4). Variation partitioning analysis (Fig. 5, C and E) further revealed that for terraces in humid regions, ligand exchange interaction was the dominant pathway controlling SPR (10.5%) and SOC stock (22.4%), while the role of the other pathways (i.e., cation bridging, SOC chemistry) are negligible (0 to 4.9%). Collectively, the increased moisture availability and transport in humid climates promote the chemical weathering and create an abundance of soil metal oxides that can directly protect the SOC in terraces of humid regions.

In contrast, for terraces in arid regions, the specific moisture and temperature conditions limit soil formation and leaching, resulting in neutral to alkaline soils enriched with base cations (e.g., Ca_ex_, Mg_ex_, and K_ex_) or highly crystallized metal oxides (ΣAl_d_, Fe_d_, and Mn_d_) (i.e., Fig. 4). We found that for terraces in arid regions, the most influential explanatory variables for SPR and SOC stock were exchangeable cations (K_ex_, Mg_ex_, or Ca_ex_, TRB), silt and clay fractions, and terrace age (*P* < 0.01; Fig. 4). This was also supported by the linear mixed-effect models showing that the most variability in SPR was captured by the component representing exchangeable cations and organic metal complexes (i.e., RC3; Fig. 5A). Polyvalent Ca and Mg cations create electrostatic bridging between the mineral surfaces and anionic or polar functional groups of organic matter, making cation bridging as an important SOC protection pathway in arid climates (*26*, *28*). As a result, for terraces in arid regions, the contribution of cation bridging was significantly higher (9.2% for SPR and 28.1% for SOC stock) compared to that of humid regions (<2.5%; Fig. 5, C to F). However, the bonding efficiency through cation bridges, prevalent in arid climates, is generally weaker when compared to the ligand exchange reactions predominant in humid soils (*29*). This is also supported by the higher SPR rate observed at the fill/deposition positions of terraces in arid regions compared to those in humid regions (fig. S5). Collectively, climate conditions in arid regions form soil geochemistry with weaker SOC protection potential, thereby insufficiently preserving the deposited SOC in fill/deposition positions of terrace systems. Therefore, as the terrace ages, the deposited SOC stock of terraces could be gradually mineralized, further decreasing the SOC stock of arid terraces (*9*). These results suggest a fundamental climate-dependent difference in soil geochemistry and SOC stabilization in terraced soils between humid and arid regions, which partly explains the contrasting effects of terracing on SOC stocks across climate regions.

### Effects of plant productivity

Our results showed that terrace establishment enhanced TPP compared to nonterraced controls (average ∆TPP = 31 ± 12%; fig. S6). This trend was further supported by the vegetation indexes, where 12 of the 14 study sites showed higher values of metrics reflecting plant productivity in terraces compared to nonterraced controls (fig. S7). These findings align with previous studies demonstrating that terracing practices enhance land productivity and biomass accumulation, thereby increasing plant-derived C input to soils (*1*). By converting steep slopes into a series of relatively flat surfaces, terracing reduces slope gradient, runoff, water erosion, loss of soil, and nutrients (*30*). Consequently, terracing provides multiple ecological benefits (*31*, *32*), which are reflected in the observed changes in current ∆TPP (see Materials and Methods).

The increase in TPP due to terracing (∆TPP) provides additional plant-derived C input to terraced soils, forming a fundamental foundation for changing SOC stocks in terrace systems. This factor accounted for 50% of the observed variations in ∆SOC (*R*^2^ = 0.50; fig. S6), and its effect primarily operated through indirect pathways mediated by soil geochemistry (Fig. 3 and fig. S3). This occurs because the effect of changing plant C inputs on SOC stock is constrained by soil geochemical properties (i.e., available mineral surfaces), which determine the capacity of terraced soils to sequester new plant-derived C. These relationships have been documented in studies of nonterraced soils (*19*).

### Effect of geomorphic processes

**Fig. 6.**
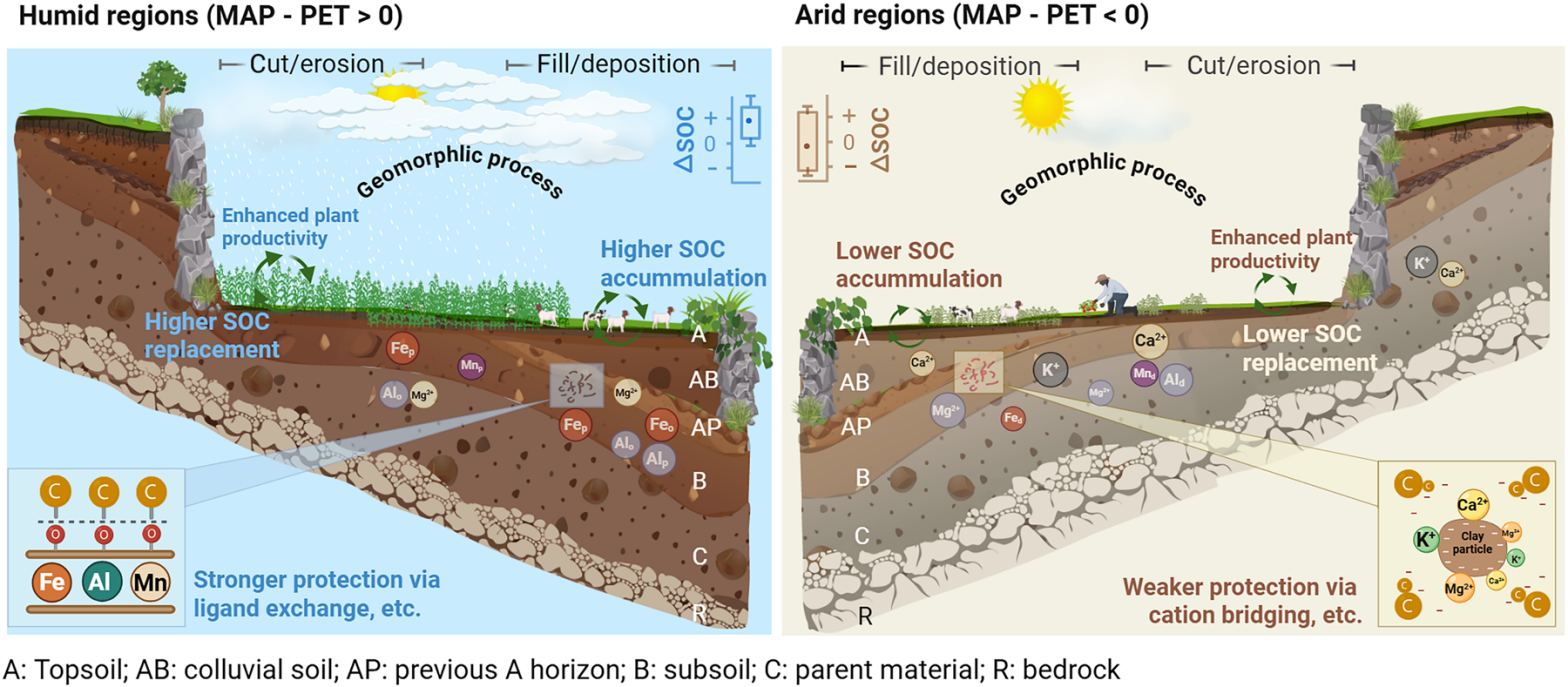
A mechanistic framework elucidating the geomorphic–biogeochemical processes regulating SOC stock changes in agricultural terraces across climate regions. The magnitude of SOC change due to terracing (∆SOC) is controlled by two key coupled C turnover-geomorphic processes: (i) the replacement of lost topsoil C at cut/erosion positions and (ii) the stabilization of buried SOC at fill/deposition positions within terrace sequences. These interplays are strongly modulated by climate through its influence on plant productivity and soil development, which in turn influences SOC replacement and stabilization within terraces, leading to significant differences in ∆SOC between humid and arid regions. Terracing enhances plant productivity and fresh C inputs, providing a fundamental foundation for changing SOC stocks. In humid regions, higher moisture availability enhances mineral weathering, forming terraced soils rich in reactive metal oxides (Fe_o_, Al_o_, Mn_o_, Fe_p_, Al_p_, and Mn_p_). This provides stronger protection for SOC in terraces. In contrast, limited moisture restricts weathering in arid regions, forming terraced soils rich in base cations (Ca^2+^, Mg^2+^, and K^+^) or highly crystallized oxides (Fe_d_, Al_d_, and Mn_d_) with weaker SOC protection. Consequently, terraces in different climate regions differ in their capacity to sequester new plant-derived C, as reflected in regional differences in SOC replacement at the cut/erosion positions and SOC accumulation at the fill/deposition of terrace sequences. This mechanistic framework explains the observed clear shift in terracing effects on SOC stocks—from consistently positive in humid regions to mixed (both positive and negative) outcomes in dry regions.

Our findings reveal that soil redistribution during terrace construction (fig. S1) functions as a “geomorphic pump,” coordinating the interaction of soil geochemistry and plant productivity to determine the magnitude and direction of terracing’s impact on SOC stocks (Figs. 1B and 6). On the one hand, SOC accumulation occurs in the fill/deposition positions during terrace formation, with a greater magnitude observed in humid regions than in arid regions (i.e., Fig. 1B). This is attributed to the stronger SOC protection in humid (i.e., ligand exchange) compared to arid (i.e., cation bridging) climates, driven by variations in soil weathering and geochemistry under contrasting climate conditions (see discussion above and Fig. 5). Consequently, the buried and deposited SOC in the fill/deposition positions of terraces in humid regions can be effectively preserved (*9*), but not in the arid regions. On the other hand, SOC lost due to topsoil removal in the cut/erosion positions during terrace excavation can be replaced by new plant-derived C inputs (*8*), with the replacement being significantly greater in humid regions compared to arid regions (see Fig. 1B). These interplays, driven by geomorphic processes during and after terracing and strongly modulated by climate-dependent biogeochemical processes, result in a significantly higher increase in SOC stocks in terraces of humid than arid regions (Fig. 1B and 6).

The replacement of lost topsoil SOC during terrace excavation is in line with the concept of dynamic replacement of C in eroding landscapes (*33*, *34*). The replacement of lost C is typically attributed to more reactive sites on mineral surfaces of subsoils that capture fresh OC inputs from plants. Ecological benefits of terracing practices enhance TPP, thereby increasing the plant-derived C input to terraced soils (as discussed in previous sections; figs. S6 and S7). This additional plant C input significantly aids the replacement of lost topsoil C in the cut/erosion position of terraces (*8*, *34*), which may contribute to the observed increase in SOC stock in agricultural terraces (Figs. 1 and (2).

The greater C replacement observed in terraces of humid regions, relative to arid regions (Fig. 1B), is attributed to the higher capacity of exposed subsurface soils to sequester fresh C under humid conditions. First, terracing enhances plant productivity (figs. S6 and S7), providing sufficient fresh organic C inputs to terraced soils (*1*, *31*, *32*). In addition, humid conditions enhance the chemical weathering of the exposed subsoil after terrace excavation (*35*), promoting the formation of new mineral-rich topsoil with higher efficiency in stabilizing C (as discussed in above sessions; Fig. 5). Thus, the lost topsoil C during terrace excavation has been completely replaced in humid regions (Fig. 1B). Second, excess moisture in humid regions may promote both the vertical and lateral transport of soluble organic compounds [e.g., dissolved organic C (DOC)] within terrace sequences. In humid climates, greater runoff enhances DOC mobility, yet terraces can intercept and retain this laterally transported DOC, while elevated soil moisture also facilitates vertical DOC infiltration and its stabilization through interactions with subsoil minerals, collectively enhancing SOC accumulation (*18*). These mechanisms all together lead to a net uptake of atmospheric C (net C sequestration) at the cut/erosion position of terraces in humid regions (Fig. 1B). However, in arid climates, limited moisture availability and specific terrace management practices under arid climates constrain the DOC transport and geochemical capacity of terraced soil to sequester new SOC (see discussion above). This limitation restricts the dynamic replacement of lost topsoil C at the cut/erosion position during terracing under arid conditions (Figs. 1B and 6). As a result, terracing practices in arid regions may cause SOC losses (e.g., Fig. 2B).

We found that the proxy for terrace age [portable optically stimulated luminescence (pOSL)] is significantly correlated with both potential soil respiration rate and SOC stock (Fig. 4B), indicating that time since construction is closely linked to SOC dynamics in terrace systems. The coupled C turnover-geomorphic processes at cut/erosional and fill/depositional positions are time dependent. On the one hand, as terraces age, the labile fraction (e.g., particulate OC) of buried SOC stock at fill/deposition positions is progressively mineralized (*9*), leading to a gradual decline in buried SOC stocks; this trend is more pronounced in arid environments, where C protection mechanisms (e.g., cation bridging) are relatively weak, consistent with the negative correlation between terrace age and SOC stock observed under arid climates (fig. S8). On the other hand, SOC replacement following topsoil loss during terrace construction exhibits a temporal lag (*15*): Prior studies indicate that SOC losses associated with initial soil removal typically require ~5 to 30 years to recover through mineral stabilization and sustained plant-derived C inputs (*8*, *11*). This mechanism explains the significant positive correlation between SOC stock and terrace age observed under humid climates (fig. S8). However, because we used bulk optically stimulated luminescence (pOSL) signal intensity as a proxy for relative terrace age (see Materials and Methods), future work using precise absolute dating (e.g., ^14^C) is warranted to more tightly constrain the role of terrace age.

### Optimizing C sequestration benefits of terracing in humid and semi-arid climates

Our research demonstrates that significant divergences in the effects of terracing on SOC stocks (*7*–*9*, *11*–*14*) are primarily driven by climate-driven processes. Pronounced differences in plant productivity and soil geochemistry between relatively humid and arid climates result in distinct couplings between SOC biogeochemical cycling and geomorphic processes occurring during terrace construction (Fig. 6). Consequently, terracing impacts on SOC shift from consistently positive in humid regions to a combination of positive and negative outcomes in arid regions. Given the widespread adoption of terracing in semi-arid regions for ecological benefits [see (*4*) and upper axis tick in Fig. 2B], targeted management strategies are urgently needed to mitigate or reverse the adverse effects of terracing on SOC storage in these regions. Our data showed that the negative effects of terracing on SOC in arid climates were mainly due to the incomplete replacement of lost topsoil C during terrace excavation (∆SOC < 0; Fig. 1B), resulting from the limited fresh C input to soil and/or insufficient available mineral surfaces. Conversely, the positive effect of terracing on SOC (∆SOC > 0) in arid regions are likely related to soil types. For instance, calcic Vertisols developed from volcanic ash can be enriched in metal oxides (fig. S9B), effectively preserving buried SOC and increasing SOC stock even under arid conditions (Fig. 1A). Soil type information from our data synthesis also showed that terraces built on more fertile or mineral-rich soils tend to enhance SOC even under arid conditions (fig. S9A). Building on these findings, we propose an integrative management framework that couples terrace engineering with measures to enhance organic C inputs or mineral weathering, aiming to mitigate SOC losses in arid regions. Specifically, coapplying crushed silicate rocks with organic matter and nutrient amendments can significantly accelerate mineral weathering and enhance soil carbon accumulation [e.g., (*36*)], relative to applications of either amendment alone. However, the effectiveness of these strategies likely varies with soil mineralogy and chemistry. For instance, silicate amendments are most effective in coarse-textured or highly weathered soils that are poor in reactive minerals (*35*, *37*). In contrast, organic and nutrient additions tend to be more effective in soils rich in Fe/Al oxides or clay minerals that facilitate mineral-organic associations and SOC stabilization (*26*). Therefore, incorporating soil property information [e.g., from SoilGrids and Harmonized World Soil Database (HWSD) maps] as a prior reference for terrace design can help optimize site-specific strategies and maximize the carbon sequestration potential of terracing.

This study provides a mechanistic framework that reveals how coupled biogeochemical-geomorphic processes govern SOC stock changes in well-drained terraces following construction across climate regions. This framework offers a scientific basis for climate- and soil-informed terrace design and management strategies to maximize C sequestration and ecological benefits of terracing practice. Considering that terraced land already covers ~5% of global cropland (*4*) and is expanding rapidly [e.g., a 24% increase in China from 2000 to 2020 (*38*)], these targeted strategies are crucial to ensure that terracing functions as a net C sink rather than a potential source, particularly in semi-arid regions.

## MATERIALS AND METHODS

### Study sites and sampling design

In this study, we selected 14 agricultural terrace landforms spanning a climatic and soil geochemical range across Europe, from a latitude of 35°N to 62°N (Norway) to a longitude of −2°W to 26°E (Greece). Mean annual temperature (MAT) and MAP ranges from 4.6° to 18.5°C and from 536 to 1657 mm, respectively. The aridity index (AI; ratio of MAP to PET) ranges from 0.32 to 2.17. Soil types include Podzols, Umbrisols, Luvisols, Vertisols, and Cambisols, developed from a wide range of parent materials, including granite, andesite, limestone, loess, etc. A detailed description of the metadata—including coordinates, soil types, parent materials, climate conditions, etc.—was provided in table S1.

The sampling sites cover semi-arid (AI = 0.2 to 0.5), semi-humid (AI = 0.5 to 0.65), and humid climate (AI > 0.65) zones, encompassing the major climatic regions where terraces are globally distributed [see global terrace distribution map from (*4*)]. Extremely arid regions (AI < 0.2) were not included in this study due to the general infeasibility of agricultural activities thus near the absence of agricultural terraces under these conditions (*2*, *4*). This study focuses on well-drained agricultural terraces, excluding paddy terraces, which remain permanently waterlogged and differ fundamentally in their hydrological and redox conditions. Across this climate-geochemical gradient, we collected 485 depth-explicit samples from 45 terraced soil profiles, excavated down to weathered bedrock (up to 190 cm depth), to measure SOC and geochemical properties (see below for details). To reliably assess the impact of terracing on SOC stocks, we additionally collected 310 SOC stock profile data from nonterraced reference points located near the 14 sampled terrace landforms using either field excavations or dataset from previous studies (see below for details). The terracing-induced changes in SOC stock (∆SOC, %) were then calculated as

$$\begin{array}{c} ∆\text{SOC}=100\times \left[\left(\text{SOC}\;\text{stoc}k_{\text{terraced}}\right.\right.\\ \left.-\text{SOC}\;\text{stoc}k_{\text{nonterraced}}\right)\left./\text{SOC}\;{\text{stock}}_{\text{nonterraced}}\right] \end{array}$$ (1)

Last, SOC stock, TPP, terrace age, and 26 soil physical, chemical, and mineralogical properties were measured, establishing the most comprehensive and consistent observation dataset to date for evaluating the impact of terracing on SOC stocks and controlling factors. A detailed description of the sampling procedures and laboratory analyses was provided below.

Of the 45 excavated terraced soil profiles, 24 were located on fill/deposition position (in front of terrace wall), while 21 were from the cut/erosion position (behind terrace wall) of terrace sequences (e.g., table S1). Each profile was cut at 5- or 10-cm-depth intervals, yielding 485 depth-explicit samples for SOC stock and detailed soil geochemical analysis. To estimate SOC stocks in nonterraced landforms, control profiles were obtained from adjacent nonterraced areas sharing the similar land use, soil type, slope, and climatic conditions. These control profiles were obtained either through field excavation, previous studies, or the Global Soil Information System (SoilGrids 2.0) database (*39*) and HWSD 2.0 (*40*). Specifically, a total of 10 control profiles were excavated down to the weathered bedrock at Smogre, Plantation Camp, Blick Mead, Gueswick, St Martens Voeren, and Choiromandres sites (see table S1); an additional 10 control profiles (as deepest as possible) were obtained for Blick Mead site from previous study (*41*); 22 control profiles with a depth of 1.5 m were taken for St Martens Voeren site (*42*); and 5 profiles reaching bedrock depth were acquired for Beloca and Terra Rosa sites, respectively (*43*); 10 control profiles were taken for Castronovo site (see table S1) (*44*). To ensure a reliable estimation of SOC stocks for the control plots, additional SOC stock profiles (10 profiles per site) were extracted for nonterraced areas from SoilGrids 2.0 and HWSD 2.0 at nine sites (see table S1). For the remaining five sites (Homolong, Skotet, Gueswick, Soave Castle, and Fornace Michelon), field measurements of SOC stocks for control plots were challenging, as nonterraced areas (under similar land use and slope conditions) near terraced landforms were absent, inaccessible due to restricted access on private property, or lacked prior field measurements. Therefore, SOC profiles for these sites were estimated from SoilGrids 2.0 and HWSD 2.0 (20 profiles per site). Estimates of ΔSOC for nonterraced sites derived from SoilGrids 2.0 and HWSD 2.0 showed reasonably good agreement with the field measurements [*R*^2^ = 0.74, *P* < 0.01, NSE (Nash-Sutcliffe efficiency) = 0.63; fig. S10; see details below]. To quantify and propagate the uncertainty of SoilGrids/HWSD-derived ΔSOC at five sites lacking field measurements for the control, we developed a Monte Carlo (MC) uncertainty-propagation framework that integrates both measurement and model-based uncertainty.

First, we fitted a linear regression between field-measured *Δ*SOC and SoilGrids/HWSD-estimated ΔSOC across all nine measured sites (excluding SM as an outlier) to characterize the systematic bias (intercept α and slope β) and residual variability (σ) between observed and gridded data. Second, for each unmeasured site, we generated *M* = 1000 MC realizations by randomly sampling SoilGrids/HWSD ΔSOC values within their uncertainty (±SE) and adding normally distributed residuals (σ). This yielded a posterior distribution of plausible ΔSOC values consistent with the empirical bias and noise structure of the measured relationship. Third, for measured sites, we generated an equivalent set of 1000 realizations by sampling from a normal distribution centered on the observed mean with its corresponding SE. Last, the complete ensemble of 1000 realizations from both measured and imputed sites were merged into a single posterior dataset and used to (i) compute site-level posterior means and 95% confidence intervals (2.5th to 97.5th percentiles) of ΔSOC (i.e., Fig. 1) and (ii) perform 1000 repeated regressions between ΔSOC and AI. The resulting posterior distributions of regression parameters (slope, *R*^2^, and *P* value) quantify how SoilGrids/HWSD uncertainty propagates into the strength and significance of the ΔSOC-climate relationship (e.g., fig. S2).

SoilGrids 2.0 contains the global predictions at 250-m spatial resolution for standard numeric soil properties (e.g., SOC stock) down to 2 m. HWSD 2.0 offers globally harmonized soil property data at 30–arc sec resolutions across seven depth layers (0 to 200 cm), compiled from regional and national soil maps. Both products represent long-term, steady-state averages of SOC stocks at the grid scale. To identify the nonterraced area in the map of SoilGrids, high-resolution digital terrain models (DTMs at 0.02-m resolution) were used to analyze the terrace geomorphological features and quantity of the land-surface metrics (*45*). The extracted features allowed the distinguish of terraced from nonterraced areas (fig. S11). Once the nonterraced areas were recognized, 10 points inside these areas were randomly selected and the values of SOC stock at six soil depths (0 to 5, 5 to 15, 15 to 30, 30 to 60, 60 to 100, and 100 to 200 cm) were extracted from SoilGrids 2.0. Similarly, using the same set of nonterraced control points, 10 SOC stock profiles per site were extracted from HWSD 2.0 for seven soil depths (0 to 20, 20 to 40, 40 to 60, 60 to 80, 80 to 100, 100 to 150, and 150 to 200 cm). The SOC stock of all layers (corresponding to the depth of excavated terraced profiles) was added up to estimate the SOC stock of the nonterraced soil profiles. More details about terrace identification using high-resolution digital terrain models and data extraction from SoilGrids 2.0 were given in text S1.

### Soil analysis

To explore the role of soil geochemistry in SOC changes in terrace systems, a diverse array of soil physicochemical properties (= 26 properties) was measured for terraced soil samples collected across Europe (*n* = 485). This encompassed soil C and nitrogen (N), SOC fractions, SPR rate, soil pedogenic oxyhydroxides (Fe, Al, and Mn), pH, soil texture, and exchangeable cations (K, Ca, Na, and Mg). Our approach involved two steps for analyzing soil physicochemical properties. First, about 20% of representative samples were chosen as calibration sample sets and analyzed by the traditional wet chemistry methods in the laboratory. This resulted in 151 calibration samples. Second, these subsets were then used to calibrate mid-infrared (MIR) spectroscopy models and predict values for the remaining 334 samples. We used a modeling approach that combined the memory-based learning with a compositional data analysis approach. This approach significantly reduces the prediction bias and ensures the reliability of the predicted values (*46*). Fair to excellent predictive models were achieved [e.g., *R*^2^ = 0.50 to 0.99, ratio of performance to deviation (RPD) = 1.4 to 8.8; table S4]. A detailed workflow for spectroscopy measurement and modeling was described by (*46*) and summarized as text S2. A detailed description of soil analysis (soil incubation, SOC fractionation, and soil physicochemical analysis) using the traditional standard laboratory methods was summarized below.

#### Soil incubation

Three replicate samples of 30 g of 2-mm sieved bulk soils (in total 151 × 3 repeats = 453 samples) were incubated in the laboratory using 380-ml sealed jars. A 10-day preincubation was carried out to avoid CO_2_ pulses caused by soil sample preparation (i.e., sieving, drying, and rewetting), and then respiration was monitored for 8 weeks while keeping temperature (20°C) and moisture (60% of soil water holding capacity) constant during the whole experiment by periodically adding demineralized water to the samples. The temperature and moisture level used during incubation was chosen to provide optimal conditions for microbial activity, thus deriving specific potential maximum heterotrophic respiration (SPR) (*47*). Respiration data were collected every 3 to 7 days to calculate SPR (μg C hour^−1^ gSOC^−1^). Headspace CO_2_ concentration was measured by an LI-830 CO_2_ gas analyzer (LI-COR Inc., the Netherlands). CO_2_ production was analyzed as the average SPR over the whole incubation period. A detailed technical description of the soil incubation experiment can be obtained from (*9*).

#### SOC fractionation

A 40 g of 5-mm sieved air-dried soils was fractionated in duplicate to obtain SOC fractions (same samples as incubation experiments). Following the fractionation scheme proposed by Six *et al.* (*48*) and simplified by Doetterl *et al.* (*19*), total SOC was fractionated into coarse particulate organic carbon (>250 μm), microaggregate-associated SOC (from 53 to 250 μm), and nonaggregated silt and clay associated SOC (<53 μm). All fractions were analyzed for total C and N using a Vario Max CN Analyzer (Elementar GmbH, Germany). The inorganic C was measured using 6 M HCl with 3% FeCl_2_·4H_2_O following (*49*). SOC content was then obtained by subtracting the inorganic C content from the total C. The C mass of each soil C fraction was calculated by multiplying the SOC concentration by the corresponding fraction mass.

#### Soil physicochemical properties

The key soil physicochemical properties were measured on the same samples as the incubation experiment: soil texture, pH_CaCl2_, the elemental composition of the soil solid phases (Fe, Mn, Al, K, Ca, Na, and Mg), base cations (K_ex_, Ca_ex_, Na_ex_, and Mg_ex_), pyrophosphate-extractable oxides (Fe_p_, Al_p_, and Mn_p_), oxalate-extractable oxides (Fe_o_, Al_o_, and Mn_o_) and dithionite-citrate-bicarbonate (DCB)–extractable oxides (Fe_d_, Al_d_, and Mn_d_). A three-step sequential extraction of pedogenic oxyhydroxides was performed on milled soils in duplicate to quantify the abundance of Al-, Fe-, and Mn-bearing fractions (*50*). First, sodium pyrophosphate at pH 10 was used for extracting organically complexed metals (Al_p_, Fe_p_, and Mn_p_) (*51*). Second, ammonium oxalate–oxalic acid at pH = 3 was used for extracting amorphous, short range–order secondary oxides and poorly crystalline aluminosilicates (Alo, Fe_o_, and Mn_o_) (*52*). Third, DCB at pH = 8 was used for extracting highly crystalline oxyhydroxides (Al_d_, Fe_d_, and Mn_d_) (*53*). In addition, total elemental compositions of Al, Fe, Mn, K, Ca, Na, and Mg were also extracted. All extracts were analyzed by inductively coupled plasma optical emission spectrometry (ICP-OES) (5100 ICP-OES Agilent Technologies, USA). Soil pH was determined with a soil/solution ratio of 1:2.5 (w/v) with 0.01 M CaCl_2_ solution using a pH meter. Soil texture was analyzed using a laser diffraction particle size analyzer (Model LS 13 320, Beckman Coulter Inc., Fullerton, USA) after ultrasonic dispersion and removal of organic matter using H_2_O_2_ (35%). For IG, MB, BL, FM, TR, and GW sites, soil bulk density (BD) was measured from an undisturbed soil sample taken with a Kubiena tin (10 cm by 10 cm by 5 cm). The weight and volume of gravels (>2 mm, if >3% in weight) were also determined to derive the BD for the fine earth (*B*D_fine_). For the remaining sites, BD was indirectly estimated from the measured soil properties (SOC, clay, silt, etc.) by calibrating pedotransfer functions proposed by Souza *et al.* (*54*)

BD=a×SOC+b×clay+c×silt+d (2)

where SOC, clay, and silt content are in %. For CL, CT, SI, and SC sites, coefficient parameters of *a*, *b*, *c*, and *d* were estimated by calibrating the Eq. 1 using the measured BD data from IG, MB, BL, FM, TR, and GW sites (obtained *R*^2^ = 0.61 to 0.70). SOC density for each soil horizon was estimated using the equation

$$\text{SOC}\;\text{density}=\frac{\text{SOC}}{100}\times {\text{BD}}_{\text{fine}}$$ (3)

where SOC density is in kg C m^−2^ cm^−1^ and SOC is in %.

The SOC stock for each soil horizon was determined by multiplying the SOC density by the thickness of the respective horizon. The SOC stock for each soil profile was then calculated as the sum of the SOC stock across all soil horizons. The changes in SOC stock due to terracing (∆SOC, %) were estimated by Eq. 1 (see above section).

### Soil burial age

The chronology of all terrace deposits is given in table S1. It was established on the basis of radiocarbon and optically stimulated luminescence (OSL) dating (*2*), as well as archeological and historical information. In addition, OSL profiling was used. As sediment (e.g., quartz and feldspar minerals) is transported by water, tillage, or wind, it will be exposed to sunlight and the trapped charge giving rise to luminescence signals zeroed. Once these sediments are deposited and subsequently buried, the absorption of ionizing radiation from the environment results in accumulation of trapped charge. Thus, the amount of absorbed energy per mass of mineral is a function of burial time. In this study, a pOSL reader was used to record the bulk OSL signal intensity. When compared to conventional OSL dating, pOSL is more efficient in terms of time, cost, and labor and can therefore be used to establish a large dataset (*55*, *56*). A detailed description of the pOSL sample collection and OSL signal measurement can be found in (*9*). pOSL was recorded during a 60-s exposure to blue light using the Scottish Universities Environmental Research Centre portable OSL meter. Consistently low photon counts at the top of the soil profiles indicate relatively young ages and high signal counts lower down in the sections, older ages, following the approach outlined in (*55*).

### FTIR spectroscopy and SOC chemistry

Considering the importance of SOC chemical composition in affecting SOC stability, we employed Fourier transform infrared (FTIR) spectroscopy to analyze SOC chemistry (*57*). Briefly, FTIR spectra were recorded using Alpha II FT-IR spectrometer (DRIFT module, Bruker Optik GmbH, Germany) over the range of 4000 and 500 cm^−1^ at a resolution of 4 cm^−1^. The spectra were baseline-corrected and converted from reflectance to absorbance. The spectral regions strongly affected by Fe and Al oxyhydroxides and silicates (i.e., <1200 cm^−1^, 1750 to 2000 cm^−1^, and >3000 cm^−1^) were excluded from SOC analysis (*58*, *59*)*.* Peaks centered around 2898 to 2976 cm^−1^ and 2839 to 2870 cm^−1^ were attributed to aliphatic C–H compounds (aliph); a larger diffuse peak centered near 1570 to 1720 cm^−1^ was interpreted as COOH compounds (acid); and peaks spanning 1500 to 1550 cm^−1^ were interpreted as aromatic C═C compounds (arom) (*58*, *60*). We followed a workflow proposed by Hodgkins *et al.* (*57*, *60*) to assess the relative abundance of the three OC groups. In brief, we semi-quantified the target compounds by isolating their representative FTIR bands and measuring peak heights. For each sample, peak positions and end points were determined individually on the basis of local minima or, when absent, on the maximum of the second derivative, followed by baseline correction between the end points. The maximum baseline-corrected absorbance was taken as the peak height and subsequently normalized by the total integrated spectral area to account for matrix and instrument effects. Ratios of arom/aliph, arom/acid, and aliph/acid were calculated to evaluate SOC chemistry. Higher arom/aliph or arom/acid ratios indicate a greater degree of decomposition, while a higher ratio of aliph/acid suggests an increase in plant versus microbially derived compounds (*58*, *61*).

### Total plant productivity

To evaluate whether changes in SOC stocks after terracing are related to the overall ecological effects of terracing practices, we used TPP as an integrative metric. We hypothesize that differences in TPP between terraces and nonterraced slopes (∆TPP) reflect the changes in plant productivity as a result of altered soil environmental conditions driven by integrative ecological effects of terracing practices, including improved land management, control erosion, retain nutrients and moisture, enhance land productivity, etc. TPP is the growing season integral, calculated as the integral sum of all daily plant phenology index values between the start and end of growing season [detailed in (*62*)]. In this study, TPP data were sourced from the high-resolution (10 m) vegetation phenology and productivity product from the European Union’s Copernicus Land Monitoring Service (*62*). In addition, three vegetation indexes—normalized difference vegetation index (NDVI), enhanced vegetation index (EVI), and soil adjusted vegetation index (SAVI)—were calculated as supplementary measures to more accurately capture the changes in TPP after terracing (*63*). NDVI, EVI, and SAVI were estimated from Sentinel-2 L2 imagery products (10-m resolution). Following the same method used for extracting SOC stock from SoilGrids 2.0, the identification and differentiation of terraced and nonterraced areas were assisted by high-resolution DTMs (fig. S11). Once the nonterraced (nearby terraces) areas were identified, 20 to 30 points were randomly selected from nonterraced areas, and the values for TPP, NDVI, EVI, and SAVI were extracted from 2017 to 2023. The changes in TPP due to terracing (∆TPP, %) were calculated

$$∆\text{TPP}=100\times \left[\left({\text{TPP}}_{\text{terraced}}-{\text{TPP}}_{\text{nonterraced}}\right)/{\text{TPP}}_{\text{nonterraced}}\right]$$ (4)

The same formula was also used to calculate ∆NDVI, ∆EVI, and ∆SAVI.

We used interannual NDVI variation (2017–2023) to evaluate the dominant land cover types between terraced and nonterraced control areas. To reduce noise in the time (daily) series data while preserving the shape of the data, we applied a Savitzky-Golay filter with a polynomial degree of 3 and a window size of 7 (*64*). Results showed that the land cover type in terraced landforms was similar with that of nonterraced controls (fig. S12).

### Additional terracing SOC data synthesis

To test the large-scale replicability of the primary climatic control on SOC changes after terracing observed from this study, we collected publications related to the effect of terrace establishment on SOC change from Web of Science, Science Direct, and Google Scholar (cutoff date: September 2025). The search used the following keywords: “terracing” or “terraces” or “terraced” and “soil organic carbon” or “carbon sequestration” or “SOC.” The inclusion criteria were as follows: (i) SOC content or stock values for both terraced and nonterraced soils (control) were reported, with the control soil having similar land use and soil type as terraced soils; (ii) terraces established less than 1 year were excluded to avoid short-term fluctuations; (iii) this study focuses on well-drained agricultural terraces; therefore, studies on permanently waterlogged paddy terraces were excluded; (iv) we focused on agricultural terraces so the gravel terrace found in fluvial context were excluded; (v) likewise, desert environment examples were also omitted from this analysis. In total, 58 publications comprising 99 paired SOC dataset met our criteria (average soil depth = 54 cm). The geographic distribution of study sites was shown on Fig. 2A. The extracted paired SOC stock data were used to calculate ∆SOC using Eq. 1 and presented in Fig. 2B. The climatic factors (MAT, MAP, and PET) were taken directly from the original studies when available; otherwise, they were extracted from global data products, i.e., MAP and MAT were taken from WorldClim2 (*65*), and PET and AI were extracted from ref. (*66*). Soil type information was extracted from the HWSD (HWSD v1.2) (*67*).

### Statistical analysis and calculations

A strong relationship between ∆SOC and climate conditions was observed (Figs. 1 and 2 and fig. S2). To further explore the importance of climate-driven processes, the dataset, which contains 485 terraced soil samples collected from Europe, was divided into two categories based on climate conditions: (i) humid terraces (MAP/PET > 1; seven study sites = 244 samples) and (ii) arid terraces (MAP/PET < 1; seven study sites = 241 samples). We used a MAP/PET threshold of 1.0 to delineate the hydrological transition where MAP equals PET, separating water-deficit from water-surplus conditions [e.g., (*18*, *37*)]. When comparing differences between climate regions, statistical analyses were conducted separately for these two categories. All the statistical analysis was performed using R 4.4.2 (R Development Core Team; www.R-project.org). Shapiro-Wilk tests were performed to check data normality, and a log transformation was conducted to address the skewed data. A *t* test was used to test the differences in soil properties between humid and arid regions. Pearson’s correlation was used to evaluate the relationship between SOC parameters (soil potential respiration and SOC stock) and terraced soil physiochemical properties.

Linear mixed-effect models [R package: lmerTest (*68*)] were used to investigate the dominant controls on SOC stock in terraced soils. A random intercept was included to address the possible clustering effects at the study site or soil profile level. The marginal *R*^2^ (*R*^2^_m_)—which quantified the variance explained by fixed effects alone—and the conditional *R*^2^ (*R*^2^_c_)—which captured the total variance explained by both fixed and random effects—were reported. A stepwise process was applied to select the best regression models for predicting SOC stock and potential respiration in terraced soils (Fig. 5, A and B). The relative importance of the variables was assessed using the standardized regression coefficient. Overall, the dataset for regression analysis consisted of 26 independent variables. We performed a rotated principal components analysis (rPCA) to reduce the data dimensionality before conducting the regression analysis. The rotated components (RCs) were used as new input variables to regression models, with a selection criterion being eigenvalue of >1 and an explained proportion of variance of >5% (*69*, *70*). We then named all RCs according to the loadings of the original variables (table S5). A threshold of *r* > 0.5 was used to decide whether an independent variable loaded into RC was used for mechanistic interpretation of the RC or not. Soil depth was added as an additional variable in regression models to avoid overinterpretation of variables because many soil properties were naturally related to soil depth.

To further identify the dominant SOC stabilization pathway in terraces of humid and arid regions, a variation partitioning analysis (VPA) (R package “vegan”) was performed (Fig. 5). The variation was portioned to three primarily SOC protection pathways: ligand exchange results primarily from metal oxides (Fe, Al, and Mn oxides) and edges of clays; cation bridging result primarily from exchangeable cations, bases, and are strongly mediated by soil pH [see (*26*, *28*, *29*)]; and SOC chemistry reflected by CN ratio and relative abundance of aromatic, aliphatic, and protonated COOH compounds [see (*71*)]. Same as linear mixed-effect models, PCA was executed to reduce data dimensionality before VPA. The first component, explaining 72 to 91% of the variation, was used for VPA following the (*72*).

To address nonlinear relationships and build predictive models for SPR and SOC stock, random forest was developed (e.g., fig. S4). Models were built using the same 26 explanatory variables as for the linear mixed-effect models. To minimize multicollinearity effects, the variance inflation factor (VIF) was computed for all independent variables, and maximal VIF was eliminated until all variables exhibited a VIP of <5 (*73*). MC cross-validation was used to assess model structure uncertainty and prevent overfitting. Data resampling was performed 100 times at a ratio of 4:1 (training to validation dataset) to ensure robustness. RMSE and *R*^2^ were estimated for all tuned models and used to evaluate model performance. To assess the importance of the identified variables for predictive models, we used the permutation variable importance measurements from the variable importance tool implemented in the “caret” package (*74*).

To compare the contributions of plant productivity and soil geochemistry to changes in SOC stock due to terracing, we conducted PLS-SEM (e.g., Fig. 3). To obtain paired data points for plant productivity and soil geochemical properties, we averaged the soil samples across depth layers and profiles, resulting in 14 paired observations. Following the best practices in PLS-SEM (*75*), we implemented additional procedures to ensure model reliability: (i) we choose PLS-SEM rather than covariance-based structural equation modeling (CB-SEM), as PLS-SEM is better suited for handling small sample size, non-normal data, and complex models, with an emphasis on predictive accuracy (*75*, *76*); (ii) we simplified the path model structure further by conducting PCA using the predictors significantly associated with ∆SOC and select the first principal component (explained >75% variation) to build the latent variables for soil geochemistry and plant productivity (*72*); and (iii) We applied bootstrapping and 10-fold cross-validation during PLS-SEM to ensure the stability of the model (*76*). The bootstrapping and 10-fold cross-validation showed a relatively small SD for *R*^2^ and path coefficients (Fig. 3). Model performance parameters indicate excellent reliability and convergent validity (table S2).

## Supplementary Materials

### This PDF file includes:

Supplementary Text S1 and S2 Figs. S1 to S12 Tables S1 to S4 References
