# Supplementary material for: Coupled geomorphic and climate-driven biogeochemical processes regulate soil organic carbon stocks in agricultural terraces
Source: Sci Adv. 2026 Feb 25;12(9):eaea8560. doi: 10.1126/sciadv.aea8560 (PMC12935031; doi:10.1126/sciadv.aea8560)
Supplement: Supplementary file 1 — Supplementary Text S1 and S2 Figs. S1 to S12 Tables S1 to S4 References [file sciadv.aea8560_sm.pdf]

Science Advances
AAAS

Supplementary Materials for

Coupled geomorphic and climate-driven biogeochemical processes regulate soil organic carbon stocks in agricultural terraces

Pengzhi Zhao *et al.*

Corresponding author: Pengzhi Zhao, pengzhi.zhao@outlook.com; Kristof Van Oost, kristof.vanoost@uclouvain.be

Sci. Adv. **12**, eaea8560 (2026)
DOI: 10.1126/sciadv.aea8560

This PDF file includes:

Supplementary Text S1 and S2
Figs. S1 to S12
Tables S1 to S4
References

Supplementary Text S1

Extracting the non–terraced SOC profiles from the SoilGrids

Remote sensing techniques have increased opportunities for mapping and characterizing terrace systems by identifying their landforms. Exploiting high–resolution topographic (HRT) technologies such as Airborne Laser Scanning (ALS) for large spatial scales, Terrestrial Laser Scanning (TLS) and Structure from Motion (SfM) photogrammetry for more detailed surveys (*77*), it is possible to produce high–resolution Digital Terrain Models (DTMs, 0.02 m resolution) that supply quantitative land–surface metrics for the analysis of terrace geomorphological features (*44*). Indeed, specific geomorphometric parameters of terrace landscapes (e.g., surface derivatives such as maximum curvature that can identify terrace edges) can be automatically extracted from DTMs using algorithms to detect terrace features (*3*). Therefore, the workflow started with remote terrace mapping at a large scale (using ALS) and then SfM–TLS data was used to produce more detailed DTMs (*2*) in different study areas. The derived DTMs and the extracted features allowed the identification of terraced and non–terraced areas along slopes (Fig. S11). Moreover, by exploiting high–resolution orthophotos of the study areas it was possible to verify the presence or absence of terraced areas. Once the non–terraced areas were recognised, 10 points inside these zones were randomly identified. For each point, the values of OCD (Organic Carbon Density) were extracted from SoilGrids – global gridded soil information (https://soilgrids.org/). SoilGrids are digital soil maps (250 m of spatial resolution) based on the global compilation of soil profile data and environmental layers, which provided OCD values in g/dm^3 for six soil depths (0–5, 5–15, 15–30, 30–60, 60–100, 100–200 cm). The OCD values for each of the 10 points belonging to the non–terraced areas were extracted using the Point Sampling Tool of QGIS software (https://plugins.qgis.org/plugins/pointsamplingtool/). Then OCD values were used to calculate SOC stock in non–terraced zones.

Supplementary Text S2

Soil spectroscopy modeling

The powdered soil samples were scanned by an Alpha II FT–IR spectrometer (DRIFT module, Bruker Optik GmbH, Germany) to determine MIR reflectance between 4000 cm^{-1} and 500 cm^{-1} at a resolution of 4 cm^{-1}. The spectra were cut to the 601 – 3996 cm^{--1} regions to remove ranges with low signal–to–noise ratios. The reflectance (*R*) was transformed into absorbance (*A*) with A = log10 (1/*R*). The first derivative with Savitzky–Golay third polynomial smoothing algorithms was applied to preprocess MIR spectra to eliminate the spectrum offset and scatter effects (*63*). The memory-based learning (MBL) algorithm was adopted to develop MIR–based prediction models for the above–mentioned soil properties.

Many soil properties involved in SOC stabilization are compositional data, typically expressed in proportions, percentages, or ppm (*45*). These data exhibit closure effects, where the sum of individual components remains constant, e.g., 100% or part of the total mass. By definition, soil texture, pedogenic oxyhydroxide, and exchangeable cations should be treated as compositional data. To reduce the prediction bias, we represented these closure effects in the spectral modeling process through additive log–ratio, centered log–ratio (*clr*), and isometric log–ratio (*ilr*) transformation methods. For the remaining soil properties (pH, C, N, SPR), the raw dataset (i.e., without log–ratio transformation) was applied. The MBL prediction model was developed using

transformed data. The predictions were back–transformed using inverse log–ratio transformation functions. The log–ratio transformation method that demonstrated the best model performance was chosen for the prediction models. Detailed workflow refers to ref. (*45*).

The reference sample set (*n*=151) was randomly divided into calibration and validation datasets at a 3:1 ratio. Using the calibration dataset, an MBL model was developed for each assessed parameter. To evaluate the model robustness, we repeated the process of random data subsetting plus subsequent model development 100 times for each modelling approach and the validation accuracy was quantified each time. The performances were evaluated by examining the root mean squared error (*RMSE*), coefficient of determination (R^2) observed against predicted values, the ratio of performance to deviation (*RPD*), and the ratio of performance to interquartile range (*RPIQ*). Following ref. (*78*), model quality was assessed by: $RPD < 1.4$ indicates a non–reliable model; $1.4 < RPD < 2$ indicates a fair model; $RPD > 2$ indicates an excellent model (Table S2).

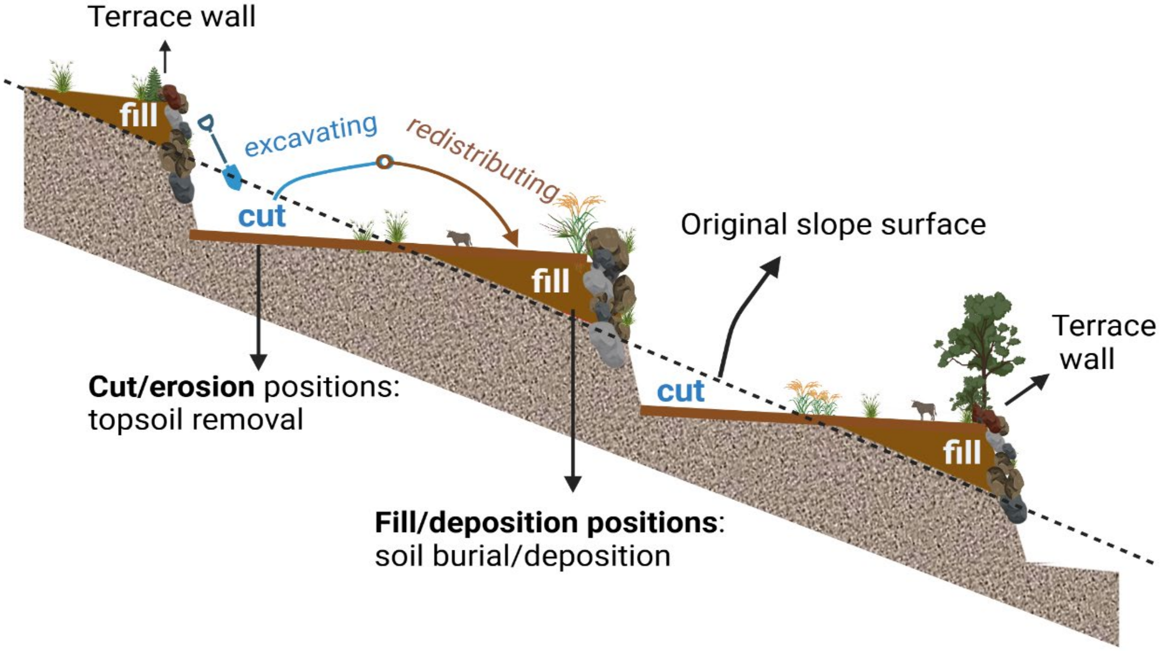

Fig. S1 Illustration of geomorphic processes occurring during terrace establishment. Terraces are typically constructed through excavating soils from the upper slope (cut position, soil and C loss) and redistributing them into the lower position of the slope (fill position, soil and C deposition/burial). Alternatively, terraces are formed through soil erosion leading to soil loss on the upper slope and soil deposition in the lower position of the terrace sequence.

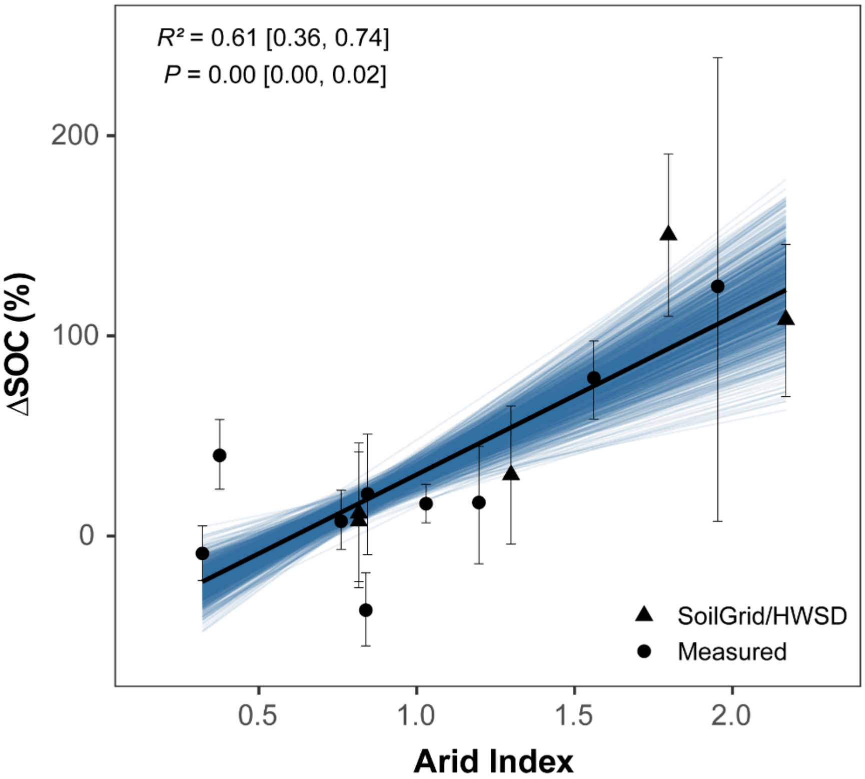

Fig. S2. Monte Carlo ensemble regressions between aridity index and terrace-induced SOC stock change (*ΔSOC*), accounting for the propagation of uncertainty from SoilGrid/HWSD-based estimates (see Methods). Thin blue lines represent 1000 individual regressions obtained from Monte Carlo realizations that integrate both measurement and model-based uncertainties. The thick black line corresponds to the regression most closely matching the ensemble mean. Points show posterior means of site-level *ΔSOC*, and vertical error bars denote 95% posterior confidence intervals (2.5^{th}–97.5^{th} percentiles). Circles indicate measured sites, and triangles indicate imputed sites. Inset statistics summarize the ensemble-mean R^2 and P-value with their 95% uncertainty ranges, illustrating how SoilGrids uncertainty influences the strength and significance of the *ΔSOC*–aridity relationship.

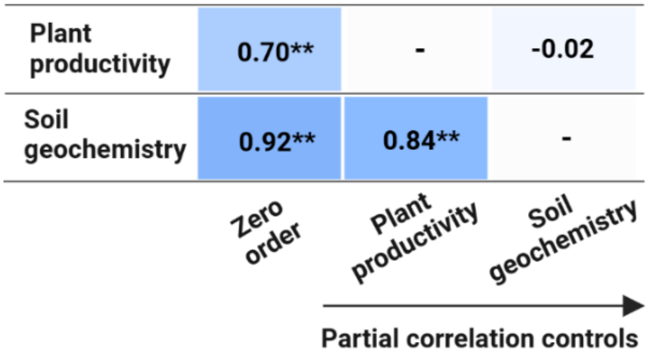

Fig. S3. Zero–order and partial correlations between terracing–induced changes in SOC stock (Δ*SOC*) and controlling factors. The difference between zero–order and partial correlations indicates the level of dependency of the correlation between a given predictor and the SOC response (see *Materials and Methods*). Colors and numbers indicate the strength and sign of correlation. ** $P < 0.01$. Results showed that controlling for soil geochemistry significantly changed the zero–order correlation between plant productivity and Δ*SOC* (from 0.70 to -0.02).

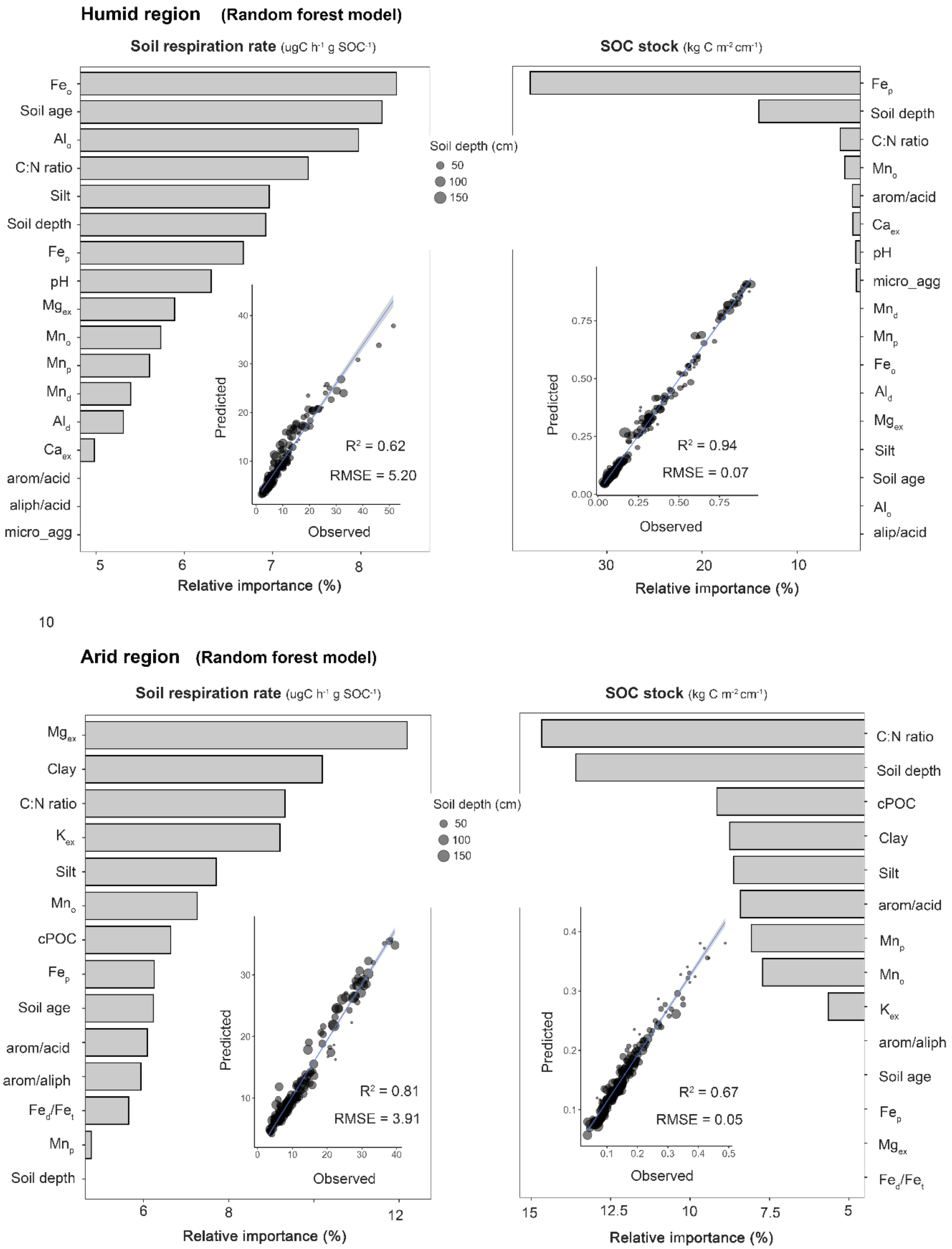

Fig. S4. Predictive random forest models and relative importance analysis for soil potential respiration rate and SOC stock of terrace samples in humid versus arid regions. *RMSE* = root mean square error; R^2 = determination coefficients. Size of point indicates the soil depth (cm). Abbreviations are explained in the caption of Fig. 4.

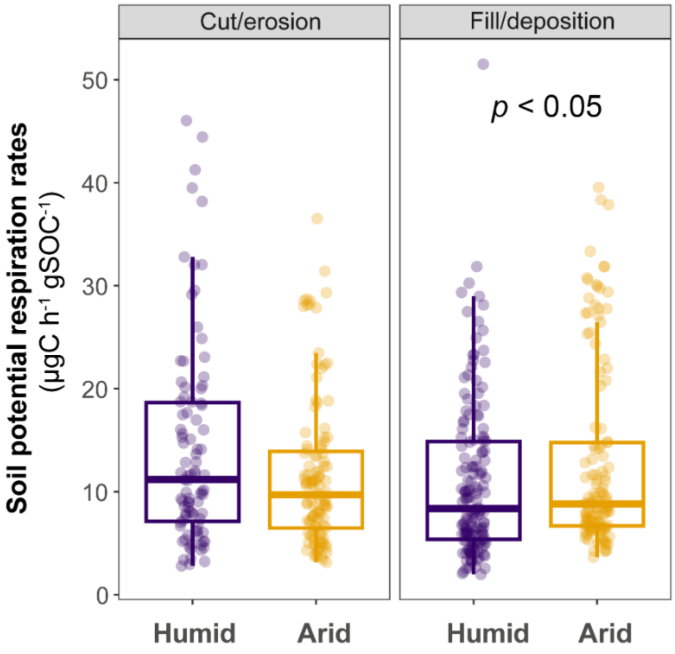

Fig. S5. Comparison of soil potential respiration rates between slope positions of terrace sequence. *Humid:* MAP/PET>1; *Arid:* MAP/PET<1. Box plots represent the first and third quartiles (box), medians (central horizontal line), upper whisker (upper vertical line) and lower whisker (lower vertical line). The significant difference between humid and arid regions for a given slope position was indicated by $P < 0.05$.

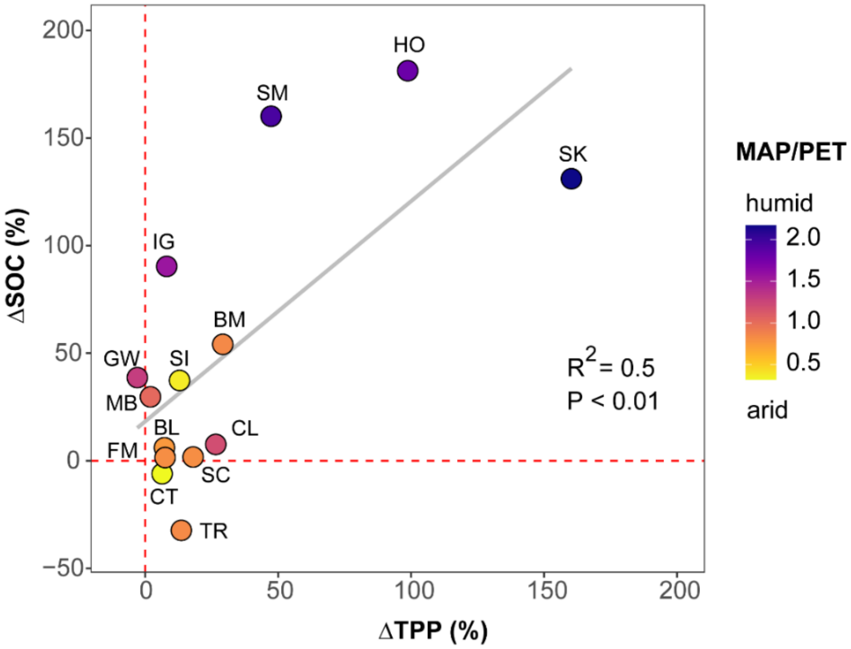

Fig. S6. Terracing–induced changes in total plant productivity (Δ*TPP*) and their relationship with ΔSOC (changes in SOC stock due to terracing). The sample site initials are provided in Table S1. $\Delta TPP = 100 \times ((TPP_{terraced} - TPP_{non-terraced})/TPP_{non-terraced})$. Colors represent the aridity index (MAP/PET). The dashed red lines mark the threshold where Δ*TPP* = 0 or Δ*SOC* = 0, distinguishing positive from negative effects of terracing on plant productivity or SOC stock, respectively. Overall, Δ*TPP* explained 50% of the variance in Δ*SOC*.

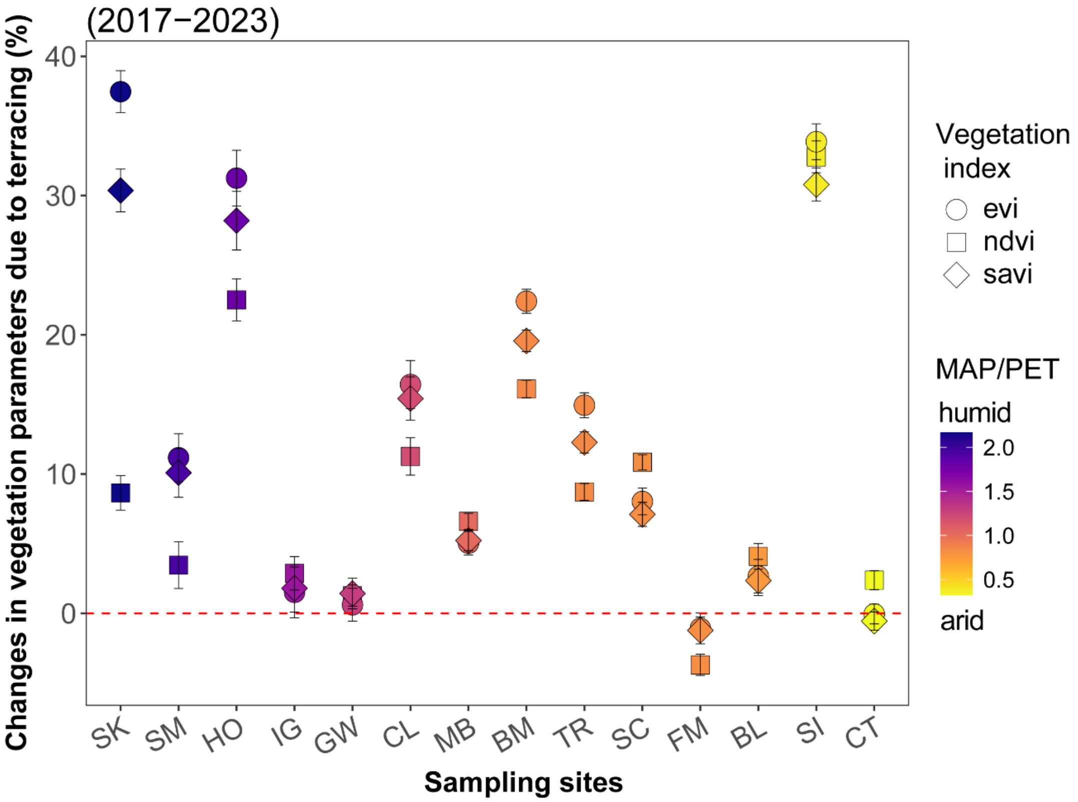

Fig. S7. Terracing–induced changes in vegetation indexes – normalized difference vegetation index ($\Delta NDVI$), enhanced vegetation index (ΔEVI) and soil adjusted vegetation index ($\Delta SAVI$). The sample site initials were provided in Table S1. $\Delta NDVI = 100 \times ((NDVI_{terraced} - NDVI_{non-terraced})/NDVI_{non-terraced})$. The same formula was used to calculate $\Delta NDVI$, $\Delta SAVI$ and ΔEVI. Colors represent the aridity index (MAP/PET). The dashed lines mark the threshold where $\Delta NDVI$, $\Delta SAVI$ or $\Delta EVI = 0$, distinguishing the positive from negative effects of terracing on vegetation indexes, e.g., $\Delta NDVI > 0$ means terracing enhances NDVI relative to non-terraced controls.

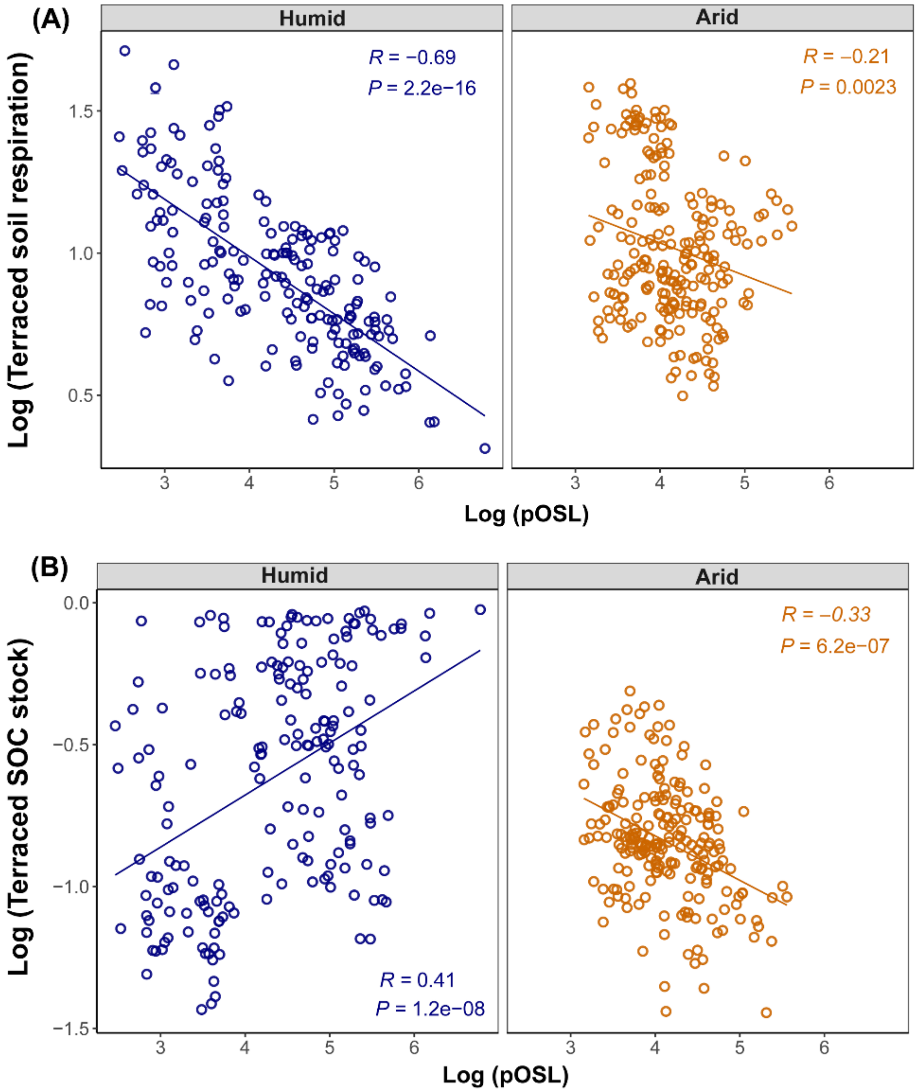

Fig. S8. Spearman correlation between terraced age proxy (pOSL) and (A) terrace soil respiration and (B) terraced SOC stock.

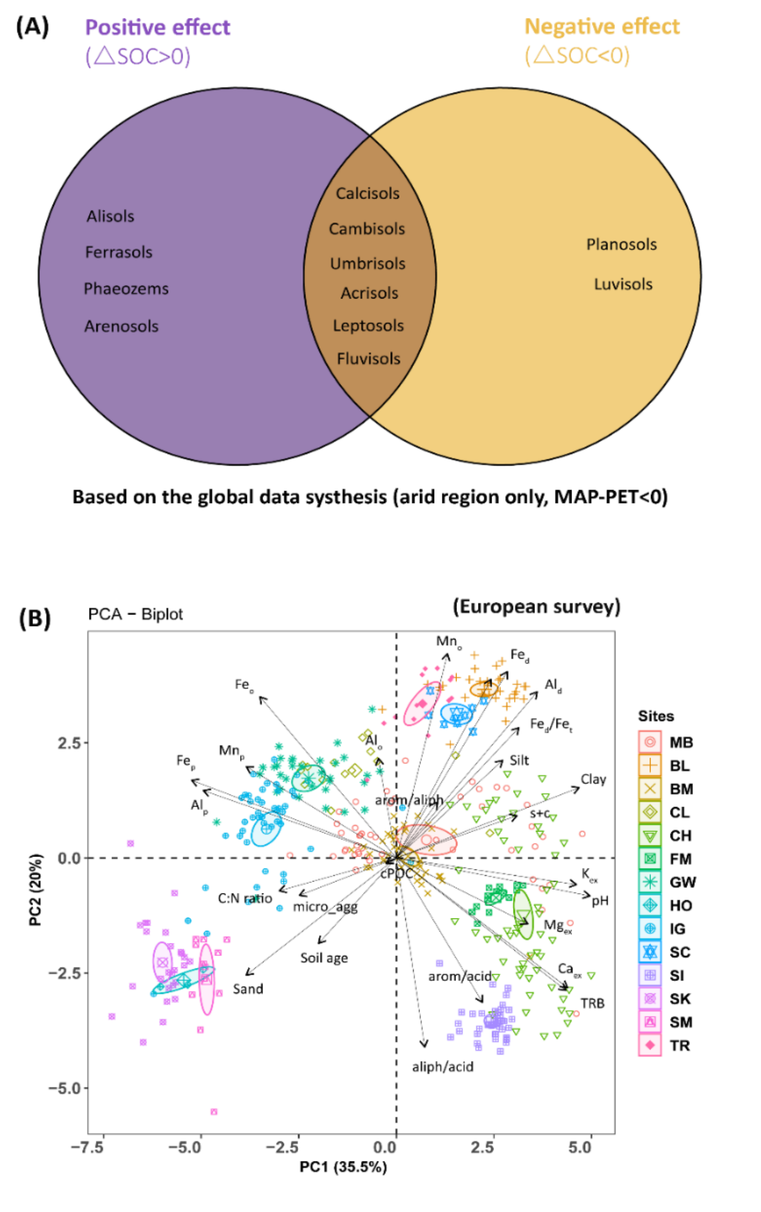

Positive effect only:
Alisols: strongly acid, high cation exchange capacity, high activity clay content in subsoil
Ferralsols: well weathered soils, high contents of iron and aluminum oxides, low amounts of available calcium or magnesium ions
Phaeozems: fertile soils with dark color, high base status
Arenosols: sandy–textured soils, lack significant soil profile development

Negative effect only:
Planosols: characterized by a subsurface layer rich in clay, poor in plant nutrients
Luvisols: moderate stage of weathering, higher clay content, high base staturation.

Fig. S9. Variation in the effect of terracing on soil organic carbon stock across different soil properties. (A) Venn diagram (based on SOC data synthesis) indicates that the mixed effects (both negative and positive) of terracing on SOC in arid regions could be partly explained by soil types on which terraces were built. Set A (cycle in purple), datapoints with a positive effect of terracing on SOC ($\Delta SOC > 0$); Set B (cycle in orange), datapoints with a negative effect of terracing on SOC ($\Delta SOC < 0$). The areas of overlap represent the intersection of set A and B, while the areas outside the circles denote elements unique to each set. Terraces built on Alisols, Arenosols, Ferralsols, and Phaeozems tend to have a positive effect on SOC, while terraces built on Luvisols and Planosols are more likely a negative effect on SOC. (**B**) Biplot for Principal component analysis (PCA) based on European terrace dataset. Calcic Vertisols (FM, BL sites in Table S1), rich in metal oxides, effectively preserve buried or deposited SOC, thereby increasing SOC stock (Fig. 1A). Site codes are given in Table S1. Abbreviations were given in Fig. 4.

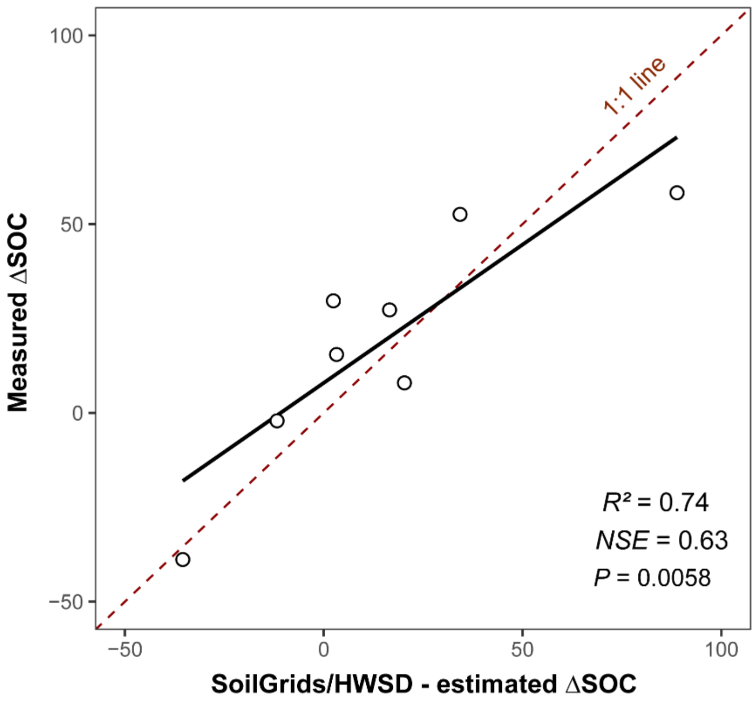

Fig. S10. Regression analysis between field-measured and SoilGrids/HWSD-estimated Δ*SOC*. Data points represent the mean Δ*SOC* of soil profiles at each site. The in-plot statistics show the coefficient of determination (R^2), Nash–Sutcliffe efficiency (*NSE*) and the *P*-value of the regression.

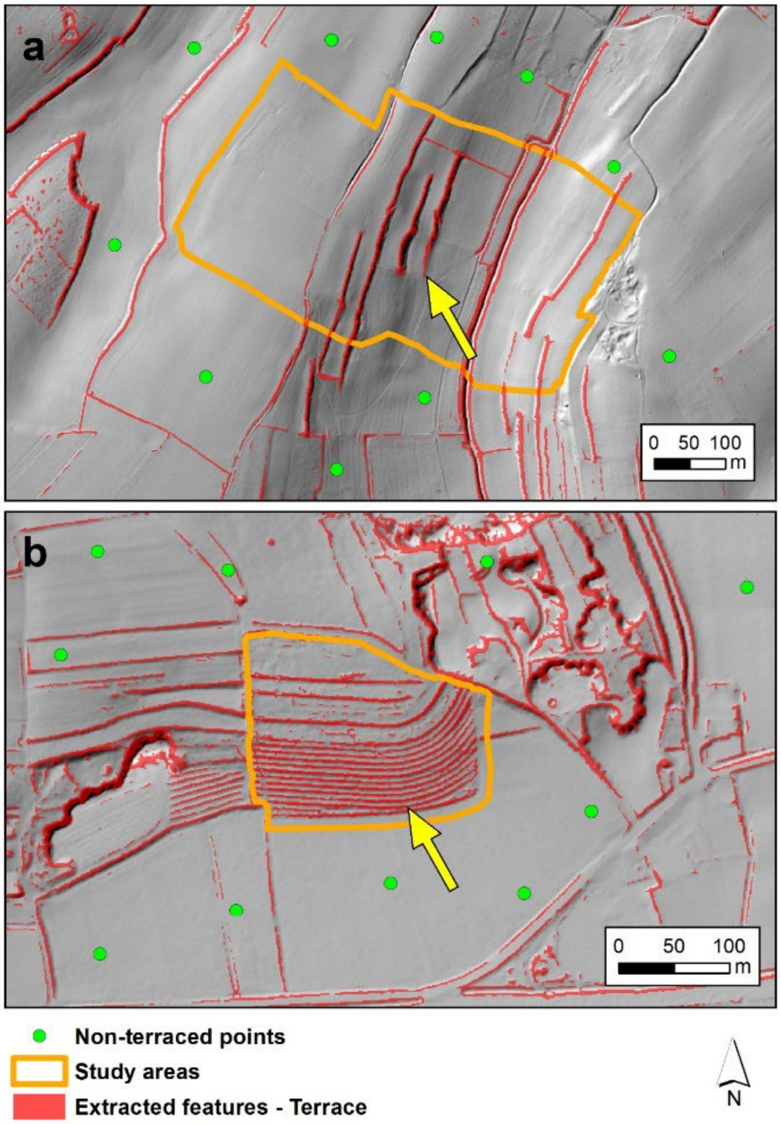

Fig. S11. Examples of the non–terraced points identified inside non–terraced areas for which the values of SOC stock were extracted from SoilGrids maps. In red colour, terrace features extracted from ALS–derived DTMs using landform curvature for the MB site in Belgium (a) and the FM site in Italy (b) (whose terraces are shown by the yellow arrow).

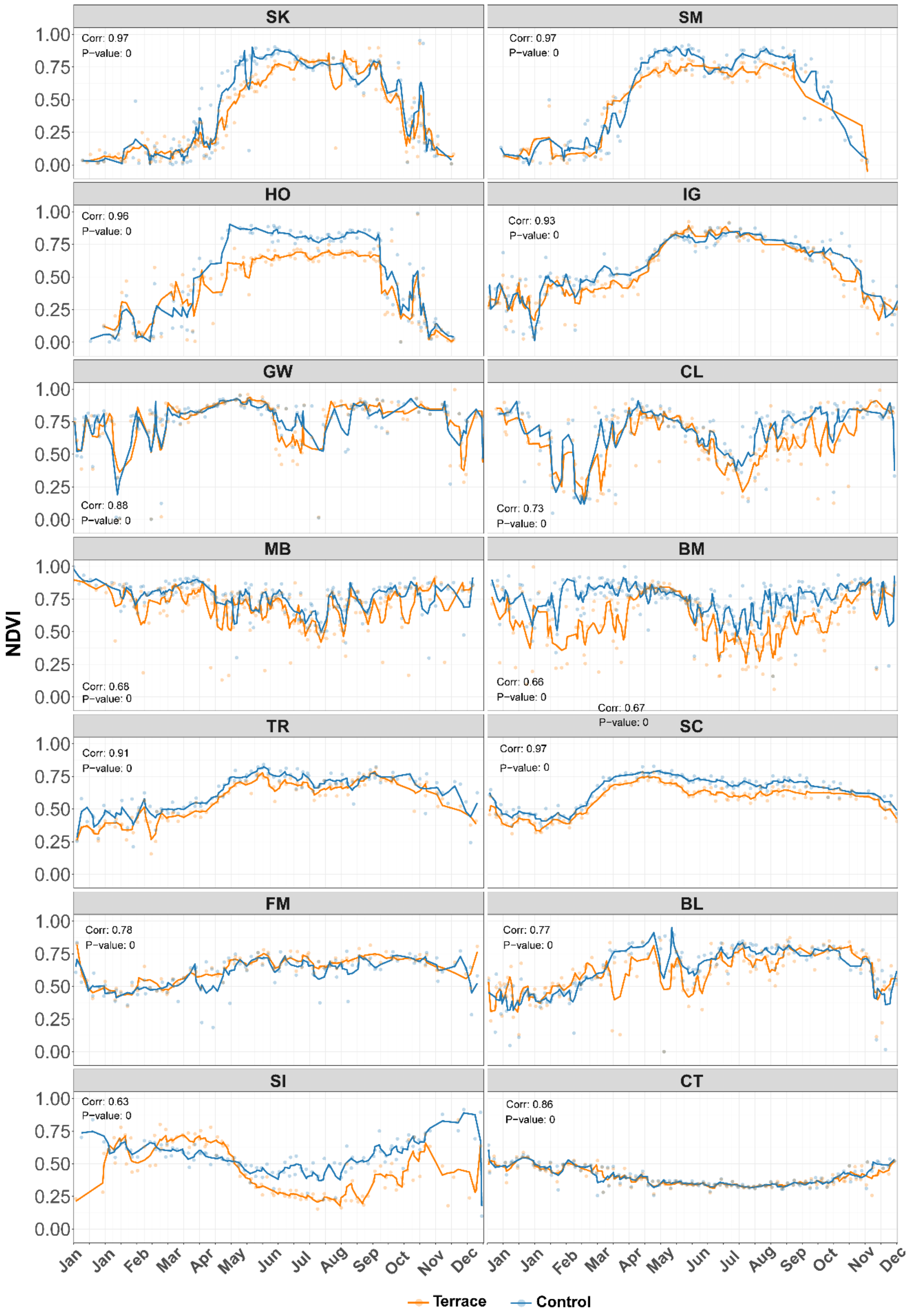

Fig. S12. Change in normalized difference vegetation index (NDVI) over time for both terraced and non–terraced control areas. The sample site initials are provided in Table S1**.** Overall, the dominant land cover type in terraced landforms was similar to that of non–terraced controls.

Table S1 Information on study sites in Europe.

Site Code	Site location	Country	Lat.	Lon.	MAT (ºC)	MAP (mm)	MAP/PET	Soil type (WRB)	Parent material	Approx. terrace age (*ref. 79*)	Terrace profiles	No. of control profiles
HO	Homolong	Norway	62.108	7.178164	4.6	1426	1.80	Podzols	Granites	19th C CE	1	10 SoilGrids + 10 HWSD
SK	Skotet	Norway	62.413094	6.901167	6.1	1657	2.17	Podzols	Granites	14-16th C CE	3	10 SoilGrids + 10 HWSD
SM	Smogre	Norway	62.235206	7.041142	6.8	1417	1.95	Podzols	Granites	1-2nd C BCE	2	1 excavated + 10 SoilGrids + 10 HWSD
IG	Plantation Camp	UK	55.44025	-1.998806	7.6	963	1.56	Umbrisols	Andesite	Bronze age to Medieval	4	2 excavated + 10 SoilGrids + 10 HWSD
BM	Blick Mead	UK	51.177283	-1.788364	9.8	689	0.84	Umbrisols	Andesite	Early Bronze age to Medieval	2	1 excavated + 10 published + 10 SoilGrids + 10 HWSD
CL	Charlton Forest	UK	50.925806	-0.730456	9.7	921	1.20	Luvisols	Loess over Chalk	Bronze age to early Medieval	2	1 excavated + 10 SoilGrids + 10 HWSD
GW	Gueswick	UK	54.584003	-1.995622	7.6	1126	1.30	Luvisols	Limestone	Late Iron age to Romano-British	4	10 SoilGrids + 10 HWSD
MB	St Martens Voeren	Belgium	50.73925	5.807881	9.4	871	1.03	Luvisols	Loess over Chalk	Iron age to Post-Medieval	6	3 excavated + 22 published + 10 HWSD
BL	Beloca	Italy	45.544264	11.204681	10.4	759	0.76	Calcic Grumic Vertisols	Volcanic or Limestone	Roman (200-400 CE)	4	10 SoilGrids + 5 published + 10 HWSD
FM	Fornace Michelon	Italy	45.411228	11.264261	13.6	871	0.82	Calcic Vertisol	Volcanic or Limestone	15-19th C CE	3	10 SoilGrids + 10 HWSD
TR	Terra Rosa	Italy	45.475514	11.316692	12.9	899	0.84	Calcic Grumic Vertisols	Volcanic or Limestone	15-19th C CE	2	10 SoilGrids + 5 published + 10 HWSD
SC	Soave Castle	Italy	45.420278	11.26075	13.3	886	0.82	Calcic Vertisols	Limestone	15-19th C CE	3	10 SoilGrids + 10 HWSD
SI	Castronovo	Italy	37.675664	13.602003	14.2	536	0.38	Cambisols	Limestone	13th C CE-modern	3	10 published + 10 SoilGrid + 10 HWSD
CT	Choiromandres	Greece	35.070903	26.238144	18.5	591	0.32	Cambisols	Limestone	c. 4000-1100 BCE	6	2 excavated + 10 SoilGrids + 10 HWSD

MAP = mean annual precipitation (mm); PET = potential evapotranspiration (mm); MAT = mean annual temperature (°C).

Table S2 Summary of model performance parameters derived from MIR spectra models.

Soil parameters	Unit	R^2	RPD	RPIQ	RMSE
Al_p	mg/kg	0.95	4.3	3.2	4.96
Al_o	mg/kg	0.65	1.6	1.0	2.45
Al_d	mg/kg	0.69	1.8	1.7	8.64
Fe_p	mg/kg	0.85	2.6	2.1	4.07
Fe_o	mg/kg	0.71	1.9	2.4	3.65
Fe_d	mg/kg	0.55	1.5	2.0	8.63
Mn_p	mg/kg	0.68	1.7	1.6	6.67
Mn_o	mg/kg	0.73	2.0	2.4	9.90
Mn_d	mg/kg	0.50	1.4	2.0	3.95
Na_{ex}	mg/kg	0.74	2.1	2.0	0.50
K_{ex}	mg/kg	0.86	2.7	4.3	3.28
Ca_{ex}	mg/kg	0.92	3.9	6.3	7.17
Mg_{ex}	mg/kg	0.91	3.4	5.7	5.83
cPOM	%	0.50	1.4	1.9	8.23
micro_agg	%	0.49	1.4	1.8	8.04
s+c	%	0.72	1.9	2.8	5.57
Clay	%	0.84	2.5	2.7	3.45
Silt	%	0.56	1.5	2.1	7.87
Sand	%	0.69	1.8	2.6	9.21
SOC	%	0.91	3.4	2.4	0.83
N	%	0.89	3.0	2.4	0.07
SPR	$\mu gC\ h^{-1}\ gsoil^{-1}$	0.79	2.1	1.9	110
TRB	cmol/kg	0.99	8.8	9.1	33
pH_{CaCl2}	-	0.93	3.9	7.4	0.40

R^2, coefficient of determination; *RMSE*, root mean square error; *RPD*, ratio of performance to deviation; *RPIQ*, ratio of performance to interquartile range. According to Chang et al. (*11*) we judge the model quality by: *RPD* < 1.4 indicates a non–reliable model; 1.4 < *RPD* < 2 indicates a fair model; *RPD* > 2 indicates an excellent model. Abbreviations are explained in the caption to Fig. 4.

Table S3. Rotated principal components (RCs) retained with a threshold eigenvalues > 1, cumulative variance explained > 80% and proportional variance > 5%.

Rotated component	RC3	RC4	RC2	RC5	RC1
**Eigenvalue**	**7.24**	**4.31**	**3.11**	**2.91**	**2.09**
**Variance explained (%)**	**31**	**19**	**14**	**13**	**9**
**Cumulative variance (%)**	**31**	**50**	**64**	**76**	**85**
Mechanistic interpretation	Exchangeable cations + organic complex Al, Fe, Mn	Highly crystalline Al, Fe, Mn	Aggregation + clay minerals	Soil burial age + texture	Poorly crystalline Al, Fe + C:N ratio
C:N ratio	-0.29	-0.26	-0.20	-0.27	**0.62**
terrace age (pOSL)	-0.17	-0.19	0.25	**-0.84**	0.16
cPOC	-0.05	-0.03	-0.05	0.12	0.08
micro_agg	-0.14	-0.14	**-0.72**	-0.08	-0.05
s+c	0.21	0.21	**0.85**	-0.07	0.00
Clay	0.48	**0.52**	**0.59**	0.13	-0.04
Silt	0.09	0.15	0.29	**0.80**	-0.1
Sand	-0.23	-0.36	**-0.61**	**-0.53**	-0.11
arom/aliph	0.02	0.09	-0.13	0.48	0.02
alip/acid	0.42	-0.42	0.21	**-0.51**	-0.06
arom/acid	**0.51**	-0.36	0.08	0.01	-0.06
K_{ex}	**0.78**	0.17	0.34	0.17	0.06
Ca_{ex}	**0.99**	-0.02	-0.01	0.01	0.02
Mg_{ex}	**0.62**	-0.04	**0.58**	-0.15	0.28
pH_{CaCl2}	**0.86**	0.27	0.11	0.17	-0.06
TRB	**0.96**	-0.05	0.14	-0.06	0.06
Al_p	**-0.83**	-0.18	-0.09	-0.20	0.37
Fe_p	**-0.90**	-0.14	-0.22	-0.14	0.24
Mn_p	**-0.71**	-0.01	-0.08	-0.07	0.21
Al_o	-0.15	0.18	0.24	0.01	**0.88**
Fe_o	**-0.74**	0.27	-0.10	-0.04	**0.51**
Mn_o	-0.20	**0.81**	0.12	0.21	-0.20
Al_d	0.19	**0.56**	0.37	**0.60**	0.10
Fe_d	0.11	**0.87**	0.30	0.13	0.21
Mn_d	0.11	**0.91**	0.13	0.05	0.22
Fe_d/Fe_t	0.21	**0.77**	-0.06	0.30	-0.37

Parallel analysis was performed to confirm the retained RC. The upper part of the table shows eigenvalues, individual and cumulative variance and mechanistic interpretation of specific RCs. Variable loadings of Pearson's $r > 0.5$ and < -0.5 are highlighted with bold + underlined font type. Note that after varimax rotation, component labels (RC) do not automatically correspond to the order of explained variance; the RCs were rearranged here in decreasing order of explained variance for clarity. ΣAl_p, Fe_p, Mn_p = sum of organically complexed Al, Fe, Mn oxides; ΣAl_o, Fe_o, Mn_o = sum of poorly crystalline Al, Fe, Mn oxides; ΣAl_d, Fe_d, Mn_d = sum of highly crystalline Al, Fe, Mn oxides; Fe_d/Fe_t = ratio of crystalline Fe oxide to total Fe. micro_agg = microaggregated OC (250–53 mm); s+c = free silt & clay associated OC (< 53 mm). arom/aliph = ratio of aromatic to aliphatic compound; aliph/acid = ratio of aliphatic to protonated COOH compounds, arom/acid = ratio of aromatic to protonated COOH compounds. TRB = total reserve in bases. K_{ex}, Ca_{ex}, Mg_{ex} = exchangeable cations of K^+, Ca^{2+}, Mg^{2+}.

Table S4 Parameters to evaluate the PLS–SEM.

	Path	Original Est.	Bootstrap Mean	Bootstrap SD	2.5% CI	97.5% CI
	Δ*TPP* -> Geochemistry	0.765	0.751	0.144	0.265	0.898
	Δ*TPP* -> Δ*SOC*	0.702	0.715	0.136	0.347	0.906
**Bootstrapped HTMT**	Geochemistry -> Δ*SOC*	0.924	0.922	0.046	0.809	0.981
						**R^2 Soil geochemistry** = 0.59
						**R^2 Δ*SOC*** = 0.86

HTMT (Heterotrait–Monotrait Ratio) typically < 0.90. Please refer to Hair et al. (*80*) for more information.

REFERENCES

1. W. Wei, D. Chen, L. Wang, S. Daryanto, L. Chen, Y. Yu, Y. Lu, G. Sun, T. Feng, Global synthesis of the classifications, distributions, benefits and issues of terracing. *Earth-Sci. Rev.* **159**, 388–403 (2016).

2. A. G. Brown, D. Fallu, K. Walsh, S. Cucchiaro, P. Tarolli, P. Zhao, B. R. Pears, K. van Oost, L. Snape, A. Lang, R. M. Albert, I. G. Alsos, C. Waddington, Ending the Cinderella status of terraces and lynchets in Europe: The geomorphology of agricultural terraces and implications for ecosystem services and climate adaptation. *Geomorphology* **379**, 107579 (2021).

3. P. Tarolli, F. Preti, N. Romano, Terraced landscapes: From an old best practice to a potential hazard for soil degradation due to land abandonment. *Anthropocene* **6**, 10–25 (2014).

4. Y. Li, F. Tian, M. Zhang, H. Zeng, S. Ahmed, X. Qin, Y. Liu, L. Wang, R. Fan, B. Wu, A 10-meter global terrace mapping using sentinel-2 imagery and topographic features with deep learning methods and cloud computing platform support. *Int. J. Appl. Earth Obs. Geoinf.* **139**, 104528 (2025).

5. B. Cao, L. Yu, V. Naipal, P. Ciais, W. Li, Y. Zhao, W. Wei, D. Chen, Z. Liu, P. Gong, A 30 m terrace mapping in China using Landsat 8 imagery and digital elevation model based on the Google Earth Engine. *Earth Syst. Sci. Data* **13**, 2437–2456 (2021).

6. T. Whitelaw, The ethnoarchaeology of recent rural settlement and land use in northwest Keos. *Landscape archaeology as long-term history: Northern Keos in the Cycladic Islands* **403–454**, (1991).

7. X. Gao, W. Li, A. Salman, R. Wang, L. Du, L. Yao, Y. Hu, S. Guo, Impact of topsoil removal on soil CO_2 emission and temperature sensitivity in Chinese Loess Plateau. *Sci. Total Environ.* **708**, 135102 (2020).

8. M. De Blécourt, V. M. Hänsel, R. Brumme, M. D. Corre, E. Veldkamp, Soil redistribution by terracing alleviates soil organic carbon losses caused by forest conversion to rubber plantation. *For. Ecol. Manage.* **313**, 26–33 (2014).

9. P. Zhao, D. J. Fallu, S. Cucchiaro, P. Tarolli, C. Waddington, D. Cockcroft, L. Snape, A. Lang, S. Doetterl, A. G. Brown, K. Van Oost, Soil organic carbon stabilization mechanisms and temperature sensitivity in old terraced soils. *Biogeosciences* **18**, 6301–6312 (2021).

10. K. Li, J. Yang, J. Wang, Z. Wang, Y. Zeng, P. Borrelli, K. Hubacek, Y. Hu, B. Xu, N. Fang, C. Zeng, Z. Zhou, Z. Shi, Human-altered soil loss dominates nearly half of water erosion in China but surges in agriculture-intensive areas. *One Earth* **7**, 2008–2018 (2024).

11. D. Chen, W. Wei, S. Daryanto, P. Tarolli, Does terracing enhance soil organic carbon sequestration? A national-scale data analysis in China. *Sci. Total Environ.* **72**, 1137751 (2020).

12. C. Zhang, G. Liu, S. Xue, C. Sun, Soil organic carbon and total nitrogen storage as affected by land use in a small watershed of the Loess Plateau, China. *Eur. J. Soil Biol.* **54**, 16–24 (2013).

13. M. C. Ramos, R. Cots-Folch, J. A. Martínez-Casasnovas, Effects of land terracing on soil properties in the Priorat region in Northeastern Spain: A multivariate analysis. *Geoderma* **142**, 251–261 (2007).

14. J. Hamdan, C. P. Burnham, B. Ruhana, Degradation effect of slope terracing on soil quality for *Elaeis guineensis* Jacq. (oil palm) cultivation. *Land Degrad. Dev.* **11**, 181–193 (2000).

15. K. Van Oost, J. Six, Reconciling the paradox of soil organic carbon erosion by water. *Biogeosciences* **20**, 635–646 (2023).

16. P. Zhao, S. Doetterl, Z. Wang, A. M. Hoyt, E. Wang, H. Yu, L. Quijano, D. J. Fallu, A. G. Brown, J. Six, K. Van Oost, Factors controlling SOC stability in colluvial soils under contrasting climate and soil weathering conditions. *Eur. J. Soil Sci.* **73**, e13311 (2022).

17. A. A. Berhe, J. W. Harden, M. S. Torn, M. Kleber, S. D. Burton, J. Harte, Persistence of soil organic matter in eroding versus depositional landform positions. *J. Geophys. Res. Biogeosci.* **117**, doi.org/10.1029/2011JG001790 (2012).

18. K. A. Heckman, A. R. Possinger, B. D. Badgley, M. M. Bowman, A. C. Gallo, J. A. Hatten, L. E. Nave, M. D. SanClements, C. W. Swanston, T. L. Weiglein, W. R. Wieder, B. D. Strahm,

Moisture-driven divergence in mineral-associated soil carbon persistence. *Proc. Natl. Acad. Sci. U.S.A.* **120**, e2210044120 (2023).

19. S. Doetterl, A. Stevens, J. Six, R. Merckx, K. Van Oost, M. Casanova Pinto, A. Casanova-Katny, C. Muñoz, M. Boudin, E. Zagal Venegas, P. Boeckx, Soil carbon storage controlled by interactions between geochemistry and climate. *Nat. Geosci.* **8**, 780–783 (2015).

20. R. A. Viscarra Rossel, J. Lee, T. Behrens, Z. Luo, J. Baldock, A. Richards, Continental-scale soil carbon composition and vulnerability modulated by regional environmental controls. *Nat. Geosci.* **12**, 547–552 (2019).

21. T. R. Underwood, I. C. Bourg, K. M. Rosso, Mineral-associated organic matter is heterogeneous and structured by hydrophobic, charged, and polar interactions. *Proc. Natl. Acad. Sci. U.S.A.* **121**, e2413216121 (2024).

22. W. Li, Z. Li, Y. Liu, X. Nie, C. Deng, G. Zhang, S. Wang, T. Xiao, H. Zheng, Reshaping of soil carbon and nitrogen contents in quincentenary ancient rice terraces: The role of both short-term abandonment and prokaryotic functional groups. *Front. Microbiol.* **13**, 1007237 (2022).

23. B. D. Hunter, J. J. Roering, L. C. R. Silva, K. C. Moreland, Geomorphic controls on the abundance and persistence of soil organic carbon pools in erosional landscapes. *Nat. Geosci.* **17**, 151–157 (2024).

24. C. R. Lawrence, M. S. Schulz, C. A. Masiello, O. A. Chadwick, J. W. Harden, The trajectory of soil development and its relationship to soil carbon dynamics. *Geoderma* **403**, 115378 (2021).

25. S. Doetterl, J. Six, B. Van Wesemael, K. Van Oost, Carbon cycling in eroding landscapes: Geomorphic controls on soil organic C pool composition and C stabilization. *Glob. Chang. Biol.* **18**, 2218–2232 (2012).

26. M. Kleber, K. Eusterhues, M. Keiluweit, C. Mikutta, R. Mikutta, P. S. Nico, Mineral-organic associations: Formation, properties, and relevance in soil environments. *Adv. Agron.* **130**, 1–140 (2015).

27. B. D. Hunter, J. J. Roering, P. C. Almond, O. A. Chadwick, M. L. Polizzotto, L. C. R. Silva, Pedogenic pathways and deep weathering controls on soil organic carbon in Pacific Northwest forest soils. *Geoderma* **436**, 116531 (2023).

28. L. Han, K. Sun, J. Jin, B. Xing, Some concepts of soil organic carbon characteristics and mineral interaction from a review of literature. *Soil Biol. Biochem.* **94**, 107–121 (2016).

29. M. V. Lützow, I. Kögel-Knabner, K. Ekschmitt, E. Matzner, G. Guggenberger, B. Marschner, H. Flessa, Stabilization of organic matter in temperate soils: Mechanisms and their relevance under different soil conditions - A review. *Eur. J. Soil Sci.* **57**, 426–445 (2006).

30. C. Deng, G. Zhang, Y. Liu, X. Nie, Z. Li, J. Liu, D. Zhu, Advantages and disadvantages of terracing: A comprehensive review. *Int. Soil Water Conserv. Res.* **9**, 344–359 (2021).

31. W. Wei, X. Feng, L. Yang, L. Chen, T. Feng, D. Chen, The effects of terracing and vegetation on soil moisture retention in a dry hilly catchment in China. *Sci. Total Environ.* **647**, 1323–1332 (2019).

32. Y. Qi, W. Wei, J. Li, C. Chen, Y. Huang, Effects of terracing on root distribution of Pinus tabulaeformis Carr. forest and soil properties in the Loess Plateau of China. *Sci. Total Environ.* **721**, 137506 (2020).

33. K. Van Oost, T. A. Quine, G. Govers, S. De Gryze, J. Six, J. W. Harden, J. C. Ritchie, G. W. McCarty, G. Heckrath, C. Kosmas, J. V. Giraldez, J. R. Marques Da Silva, R. Merckx, The impact of agricultural soil erosion on the global carbon cycle. *Science* **318**, 626–629 (2007).

34. J. W. Harden, J. M. Sharpe, W. J. Parton, D. S. Ojima, T. L. Fries, T. G. Huntington, S. M. Dabney, Dynamic replacement and loss of soil carbon on eroding cropland. *Global Biogeochem. Cycles* **13**, 885–901 (1999).

35. S. Doetterl, A. A. Berhe, C. Arnold, S. Bodé, P. Fiener, P. Finke, L. Fuchslueger, M. Griepentrog, J. W. Harden, E. Nadeu, J. Schnecker, J. Six, S. Trumbore, K. Van Oost, C. Vogel, P. Boeckx, Links among warming, carbon and microbial dynamics mediated by soil mineral weathering. *Nat. Geosci.* **11**, 589–593 (2018).

36. J. Sohng, N. W. Sokol, S. Whiteaker, R. Schmidt, I. Holzer, H. Goertzen, J. Peña, B. Z. Houlton, I. Montañez, A. O'Geen, K. Scow, Combining organic amendments with enhanced rock weathering shifts soil carbon storage in croplands. *Sci. Total Environ.* **998**, 180179 (2025).

37. E. Zhang, Y. Chen, S. Wei, C. Liu, H. Wang, B. Deng, H. Lin, X. Yang, Y. Li, X. Duan, A 30 m soil and water conservation terrace measures dataset of China from 2000 to 2020. *Earth Syst. Sci. Data* **17**, 6315–6330 (2025).

38. L. Poggio, L. M. De Sousa, N. H. Batjes, G. B. M. Heuvelink, B. Kempen, E. Ribeiro, D. Rossiter, SoilGrids 2.0: Producing soil information for the globe with quantified spatial uncertainty. *SOIL* **7**, 217–240 (2021).

39. W. Wieder, Regridded harmonized world soil database v1. 2. ORNL Distributed Active Archive Center (DAAC) dataset (2014); 10.3334/ORNLDAAC/1247.

40. E. L. Fry, J. Savage, W. J. Pritchard, R. D. Bardgett, R. F. Pywell, J. M. Bullock, Total carbon and nitrogen stocks across a land use gradient on Salisbury Plain in June 2014. (NERC Environmental Information Data Centre, 2016); https://doi.org/10.5285/58709d9b-2b52-4f5d-8f3b-49354e664aea.

41. J. Meersmans, B. van Wesemael, E. Goidts, M. Van Molle, S. De Baets, F. De Ridder, Spatial analysis of soil organic carbon evolution in Belgian croplands and grasslands, 1960–2006. *Glob. Chang. Biol.* **17**, 466–479 (2011).

42. G. Pellis, T. Chiti, A. Rey, J. C. Yuste, C. Trotta, D. Papale, The ecosystem carbon sink implications of mountain forest expansion into abandoned grazing land: The role of subsoil and climatic factors. *Sci. Total Environ.* **672**, 106–120 (2019).

43. A. Novara, P. Pereira, A. Santoro, Y. Kuzyakov, T. La Mantia, Effect of cactus pear cultivation after Mediterranean maquis on soil carbon stock, δ13C spatial distribution and root turnover. *Catena* **118**, 84–90 (2014).

44. S. Cucchiaro, G. Paliaga, D. J. Fallu, B. R. Pears, K. Walsh, P. Zhao, K. Van Oost, L. Snape, A. Lang, A. G. Brown, P. Tarolli, Volume estimation of soil stored in agricultural terrace systems: A geomorphometric approach. *Catena* **207**, 105687 (2021).

45. P. Zhao, D. J. Fallu, B. R. Pears, C. Allonsius, J. J. Lembrechts, S. Van de Vondel, F. J. R. Meysman, S. Cucchiaro, P. Tarolli, P. Shi, J. Six, A. G. Brown, B. van Wesemael, K. Van Oost, Quantifying soil properties relevant to soil organic carbon biogeochemical cycles by infrared spectroscopy: The importance of compositional data analysis. *Soil Tillage Res.* **231**, 105718 (2023).

46. C. K. Paull, W. Ussler, P. J. Mitts, D. W. Caress, G. J. West, Discordant 14C-stratigraphies in upper Monterey Canyon: A signal of anthropogenic disturbance. *Mar. Geol.* **233**, 21–36 (2006).

47. J. Six, E. T. Elliott, K. Paustian, J. W. Doran, Aggregation and soil organic matter accumulation in cultivated and native grassland soils. *Soil Sci. Soc. Am. J.* **62**, 1367–1377 (1998).

48. L. A. Sherrod, G. Dunn, G. A. Peterson, R. L. Kolberg, Inorganic carbon analysis by modified pressure-calcimeter method. *Soil Sci. Soc. Am. J.* **66**, 299–305 (2002).

49. J. W. Stucki, B. A. Goodman, U. Schwertmann, Eds., *Iron in Soils and Clay Minerals* (Springer Science & Business Media, 2012), vol 217.

50. C. L. Bascomb, Distribution of Pyrophosphate-Extractable Iron and Organic Carbon in Soils of Various Groups. *J. Soil Sci.* **19**, 251–268 (1968).

51. R. A. Dahlgren, Quantification of allophane and imogolite," in *Quantitative Methods in Soil Mineralogy*, J. E. Amonette, L. W. Zelazny, Eds. (SSSA Miscellaneous Publication, 1994), p. 430.

52. O. P. Mehra, M. L. Jackson, Iron Oxide removal from soils and clays by a dithionite-citrate system buffered with sodium bicarbonate. *Clays Clay Miner.* **7**, 317–327 (1958).

53. E. de Souza, E. I. F. Filho, C. E. G. R. Schaefer, N. H. Batjes, G. R. dos Santos, L. M. Pontes, Pedotransfer functions to estimate bulk density from soil properties and environmental covariates: Rio Doce basin. *Sci. Agric.* **73**, 525–534 (2016).

54. E. Muñoz-Salinas, P. Bishop, D. C. Sanderson, J. J. Zamorano, Interpreting luminescence data from a portable OSL reader: Three case studies in fluvial settings. *Earth Surf. Process. Landf.* **36**, 651–660 (2011).

55. N. Porat, G. I. López, N. Lensky, R. Elinson, Y. Avni, Y. Elgart-Sharon, G. Faershtein, Y. Gadot, Using portable OSL reader to obtain a time scale for soil accumulation and erosion in archaeological terraces, the Judean Highlands. *Israel. Quat. Geochronol.* **49**, 65–70 (2019).

56. S. B. Hodgkins, M. M. Tfaily, C. K. McCalley, T. A. Logan, P. M. Crill, S. R. Saleska, V. I. Rich, J. P. Chanton, Changes in peat chemistry associated with permafrost thaw increase greenhouse gas production. *Proc. Natl. Acad. Sci. U.S.A.* **111**, 5819–5824 (2014).

57. S. J. Hall, A. A. Berhe, A. Thompson, Order from disorder: Do soil organic matter composition and turnover co-vary with iron phase crystallinity? *Biogeochemistry* **140**, 93–110 (2018).

58. S. J. Parikh, K. W. Goyne, A. J. Margenot, F. N. D. Mukome, F. J. Calderón, Soil chemical insights provided through vibrational spectroscopy. *Adv. Agron.* **126**, 1–148 (2014).

59. S. B. Hodgkins, C. J. Richardson, R. Dommain, H. Wang, P. H. Glaser, B. Verbeke, B. R. Winkler, A. R. Cobb, V. I. Rich, M. Missilmani, N. Flanagan, M. Ho, A. M. Hoyt, C. F. Harvey, S. R. Vining, M. A. Hough, T. R. Moore, P. J. H. Richard, F. B. De La Cruz, J. Toufaily, R. Hamdan, W. T. Cooper, J. P. Chanton, Tropical peatland carbon storage linked to global latitudinal trends in peat recalcitrance. *Nat. Commun.* **9**, 3640 (2018).

60. M. S. Demyan, F. Rasche, E. Schulz, M. Breulmann, T. Müller, G. Cadisch, Use of specific peaks obtained by diffuse reflectance Fourier transform mid-infrared spectroscopy to study the composition of organic matter in a Haplic Chernozem. *Eur. J. Soil Sci.* **63**, 189–199 (2012).

61. Copernicus Land Monitoring Service, High Resolution Vegetation Phenology and Productivity (HRVPP), Seasonal Trajectories and VPP parameters. (2021).

62. M. E. Fadl, M. A. E. AbdelRahman, A. I. El-Desoky, Y. A. Sayed, Assessing soil productivity potential in arid region using remote sensing vegetation indices. *J. Arid Environ.* **222**, 105166 (2024).

63. A. Savitzky, M. J. E. Golay, Smoothing and differentiation of data by simplified least squares procedures. *Anal. Chem.* **36**, 1627–1639 (1964).

64. S. E. Fick, R. J. Hijmans, World Clim 2: New 1-km spatial resolution climate surfaces for global land areas. *Int. J. Climatol.* **37**, 4302–4315 (2017).

65. R. J. Zomer, J. Xu, A. Trabucco, Version 3 of the global aridity index and potential evapotranspiration database. *Sci. Data* **9**, 409 (2022).

66. F. Nachtergaele, H. van Velthuizen, L. Verelst, D. Wiberg, M. Henry, F. Chiozza, Y. Yigini, E. Aksoy, N. Batjes, E. Boateng, Harmonized World Soil Database Version 2.0 (Food and Agriculture Organization of the United Nations, 2023).

67. M. G. Kramer, O. A. Chadwick, Climate-driven thresholds in reactive mineral retention of soil carbon at the global scale. *Nat. Clim. Chang.* **8**, 1104–1108 (2018).

68. A. Kuznetsova, P. B. Brockhoff, R. H. B. Christensen, lmerTestPackage: tests in linear mixed effects models. *J. Stat. Softw.* **82**, 1–26 (2017).

69. J. Gareth, W. Daniela, H. Trevor, T. Robert, *An Introduction to Statistical Learning: With Applications in R* (Springer, 2013).

70. I. T. Jolliffe, Rotation of principal components: Choice of normalization constraints. *J. Appl. Stat.* **22**, 29–35 (1995).

71. G. Angst, K. E. Mueller, K. G. J. Nierop, M. J. Simpson, Plant- or microbial-derived? A review on the molecular composition of stabilized soil organic matter. *Soil Biol. Biochem.* **156**, 108189 (2021).

72. S. Qin, D. Kou, C. Mao, Y. Chen, L. Chen, Y. Yang, Temperature sensitivity of permafrost carbon release mediated by mineral and microbial properties. *Sci. Adv.* **7**, eabe3596 (2021).

73. D. Haaf, J. Six, S. Doetterl, Global patterns of geo-ecological controls on the response of soil respiration to warming. *Nat. Clim. Chang.* **11**, 623–627 (2021).

74. M. Kuhn, Building predictive models in R using the caret package. *J. Stat. Softw.* **28**, 1–26 (2008).

75. J. F. Hair, C. M. Ringle, M. Sarstedt, PLS-SEM: Indeed a silver bullet. *J. Mark. Theory Pract.* **19**, 139–152 (2011).

76. N. F. Richter, A. A. Tudoran, Elevating theoretical insight and predictive accuracy in business research: Combining PLS-SEM and selected machine learning algorithms. *J. Bus. Res.* **173**, 114453 (2024).

77. G. Sofia, Combining geomorphometry, feature extraction techniques and Earth-surface processes research: The way forward. *Geomorphology* **355**, 107055 (2020).

78. C.-W. Chang, D. Laird, M. J. Mausbach, C. R. Hurburgh Jr., Near-infrared reflectance spectroscopy–principal components regression analyses of soil properties. *Soil Sci. Soc. Am. J.* **65**, 480–490 (2001).

79. A. G. Brown, B. Pears, S. Cucchiaro, P. Tarolli, A. Lang, P. Zhao, K. Walsh, K. Van Oost, R. M. Albert, M. Alonso-Eguiluz, L. Vokotopoulos, The Geoarchaeology of Agricultural Terraces in Europe: Construction, Resilience and Implications for Sediment Delivery. *Geoarchaeology* **40**, 70008 (2025).

80. J. F. Hair Jr, G. T. M. Hult, C. M. Ringle, M. Sarstedt, N. P. Danks, S. Ray, *Partial Least Squares Structural Equation Modeling (PLS-SEM) Using R: A Workbook* (Springer Nature, 2021).
